# N-acylsphingosine amidohydrolase 1 promotes melanoma growth and metastasis by suppressing peroxisome biogenesis-induced ROS production

**DOI:** 10.1016/j.molmet.2021.101217

**Published:** 2021-03-23

**Authors:** Parmanand Malvi, Radoslav Janostiak, Arvindhan Nagarajan, Xuchen Zhang, Narendra Wajapeyee

**Affiliations:** 1 Department of Biochemistry and Molecular Genetics, University of Alabama at Birmingham, Alabama, 35233, USA; 2 Department of Pathology, Yale University School of Medicine, New Haven, CT, 06510, USA; 3 Institute for Research in Biomedicine (IRB Barcelona), The Barcelona Institute of Science and Technology, Barcelona, 08028, Spain

**Keywords:** Melanoma, Ceramide, ASAH1, ROS, Peroxisome, Cancer therapeutics

## Abstract

**Objective:**

Metabolic deregulation is a key hallmark of cancer cells and has been shown to drive cancer growth and metastasis. However, not all metabolic drivers of melanoma are known. Based on our finding that N-acylsphingosine amidohydrolase 1 (ASAH1) is overexpressed in melanoma, the objective of these studies was to establish its role in melanoma tumor growth and metastasis, understand its mechanism of action, and evaluate ASAH1 targeting for melanoma therapy.

**Methods:**

We used publicly available melanoma datasets and patient-derived samples of melanoma and normal skin tissue and analyzed them for ASAH1 mRNA expression and ASAH1 protein expression using immunohistochemistry. ASAH1 was knocked down using short-hairpin RNAs in multiple melanoma cell lines that were tested in a series of cell culture-based assays and mouse-based melanoma xenograft assays to monitor the effect of ASAH1 knockdown on melanoma tumor growth and metastasis. An unbiased metabolomics analysis was performed to identify the mechanism of ASAH1 action. Based on the metabolomics findings, the role of peroxisome-mediated reactive oxygen species (ROS) production was explored in regard to mediating the effect of ASAH1. The ASAH1 inhibitor was used alone or in combination with a BRAFV600E inhibitor to evaluate the therapeutic value of ASAH1 targeting for melanoma therapy.

**Results:**

We determined that ASAH1 was overexpressed in a large percentage of melanoma cells and regulated by transcription factor E2F1 in a mitogen-activated protein (MAP) kinase pathway-dependent manner. ASAH1 expression was necessary to maintain melanoma tumor growth and metastatic attributes in cell cultures and mouse models of melanoma tumor growth and metastasis. To identify the mechanism by which ASAH1 facilitates melanoma tumor growth and metastasis, we performed a large-scale and unbiased metabolomics analysis of melanoma cells expressing ASAH1 short-hairpin RNAs (shRNAs). We found that ASAH1 inhibition increased peroxisome biogenesis through ceramide-mediated PPARγ activation. ASAH1 loss increased ceramide and peroxisome-derived ROS, which in turn inhibited melanoma growth. Pharmacological inhibition of ASAH1 also attenuated melanoma growth and enhanced the effectiveness of BRAF kinase inhibitor in the cell cultures and mice.

**Conclusions:**

Collectively, these results demonstrate that ASAH1 is a druggable driver of melanoma tumor growth and metastasis that functions by suppressing peroxisome biogenesis, thereby inhibiting peroxisome-derived ROS production. These studies also highlight the therapeutic utility of ASAH1 inhibitors for melanoma therapy.

## Highlights

• ASAH1 is necessary for melanoma tumor growth and metastasis.
• Transcription factor E2F1 regulates ASAH1 expression in melanoma.
• Loss of ASAH1 leads to increased peroxisome biogenesis and ROS production.
• ASAH1 inhibitor blocks melanoma and enhances the effectiveness of BRAF inhibitor.

## Graphical abstract

**Figure:**
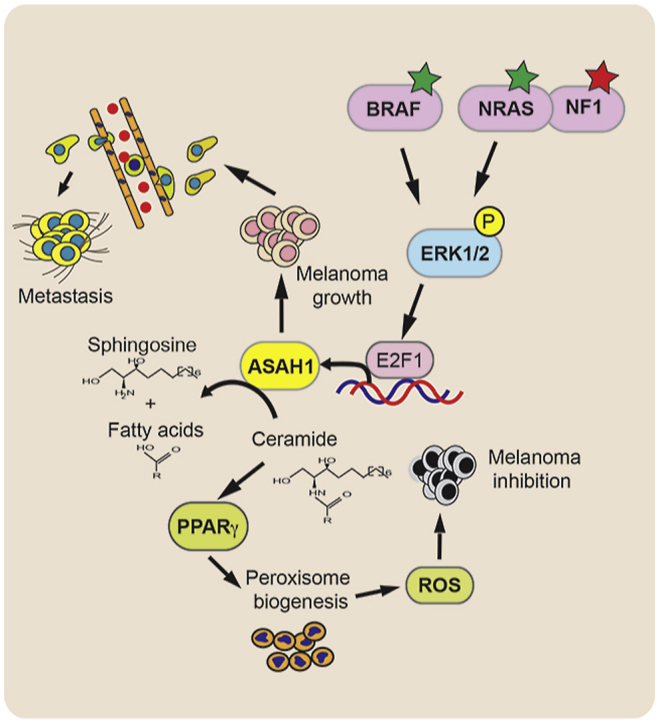

## 1 Introduction

Melanoma is the deadliest form of skin cancer and accounts for over 80% of skin cancer-related deaths [1,2]. Using genome-scale analyses, melanoma has been classified into four distinct groups based on their NRAS, BRAF, and NF1 status: NRAS-mutant, BRAF-mutant, NF1-mutant, and triple-negative (NRAS/BRAF/NF1 mutant) melanoma [3].

BRAF-mutant melanoma patients have benefited from BRAF kinase and MEK kinase inhibitor-based therapies (vemurafenib, dabrafenib, and trametinib) [4,5]. However, emerging resistance to these therapies poses a clinical challenge [[6], [7], [8]]. Similarly, while immunotherapies have proven effective in 20–30% of metastatic melanoma patients [9,10], resistance to these therapies has also emerged [11,12]. Thus, further studies are needed to determine the molecular mechanisms driving melanoma and identify new druggable pathways and targets.

Metabolic alterations are a major hallmark of cancer [[13], [14], [15], [16]]. Several metabolic alterations associated with melanoma have been identified [17,18]. However, new metabolic deregulations are frequently being revealed, demonstrating that we do not fully understand the metabolic needs of melanoma for growth and metastasis and how these needs affect therapeutic outcomes.

N-Acylsphingosine amidohydrolase 1 (ASAH1) is an acid ceramidase that converts ceramide into sphingosine and free fatty acid [19]. ASAH1 is overexpressed in many cancer types, including melanoma [20,21]. In some cancers, such as gastric cancer, ASAH1 has also been shown to predict poor prognosis [22]. ASAH1 overexpression in breast cancer is associated with lymph node metastasis [23]. However, the role of ASAH1 in melanoma growth and progression and its mechanism of action are unclear. In this report, we show that ASAH1 is required for melanoma tumor growth and metastasis. We provide evidence that ASAH1 facilitates melanoma growth by suppressing peroxisome biogenesis and peroxisome-dependent ROS production. We also demonstrate that pharmacological ASAH1 inhibition blocks melanoma growth and increases the effectiveness and therapeutic duration of a BRAF kinase inhibitor.

## 2 Materials and methods

### 2.1 Cell culture

Human melanoma cell lines A375 (BRAF mutant), M14 (BRAF mutant), MeWo (NF1-deficient), and A375-MA2 (BRAF-mutant) were obtained from the American Type Culture Collection (ATCC) and maintained as recommended by the ATCC. The YUGASP (NRAS mutant) melanoma cell line was obtained from Yale SPORE in Skin Cancer and maintained as recommended. All the cell lines were authenticated with STR analyses and routinely screened for mycoplasma.

### 2.2 shRNAs, transfection, lentivirus preparation, and stable cell line generation

All the shRNAs were obtained from Open Biosystems and are listed in Supplementary Table 1. Lentivirus particles carrying shRNA were generated by co-transfecting shRNA plasmids with lentiviral packaging plasmids pSPAX2 and pMD2.G into 293T cells using Effectene (Qiagen) according to the manufacturer's instructions. Virus was filtered using a 0.45 μm filter. Stable cell lines were generated by infecting various melanoma cell lines with shRNA lentivirus in 12-well plates followed by puromycin selection (0.2–1.5 μg/ml).

### 2.3 Anoikis assay

To evaluate anoikis, melanoma cells expressing *ASAH1* shRNA were seeded at 5 × 10^4^ cells per well in ultra-low-attachment 24-well tissue culture plates (Corning) in DMEM or RPMI as appropriate for the cell line and incubated for 7 days at 37 °C. Live cells identified based on trypan blue dye exclusion were counted using a hemocytometer. Experiments were performed in triplicate.

### 2.4 Luciferase-reporter assay

Melanoma cells were transfected with a PPRE-luciferase construct (Plasmid #1015, Addgene) carrying the indicated shRNA. After 24 h, the cells were treated with vehicle, C2 ceramide, or rosiglitazone and incubated for another 24 h. The luciferase-reporter assay was performed using a Dual-Luciferase Reporter Assay kit (Promega). Relative reporter activity was measured by determining the ratio of firefly to Renilla-luciferase activity and then normalizing values to those of cells co-transfected with non-specific shRNA.

### 2.5 Immunohistochemistry

Formalin-fixed, paraffin-embedded tissue microarray (TMA) slides containing primary skin and malignant melanoma tissues were obtained from US Biomax USA (Catalog No. ME2081 and ME803b). Briefly, following deparaffinization of the slides, antigen retrieval was performed in citrate buffer (pH 6.0) at 97 °C for 20 min using the Lab Vision PT Module (Thermo Fisher Scientific). Endogenous peroxides were blocked using hydrogen peroxide, and proteins were blocked using 0.3% BSA. The slides were incubated in ASAH1 antibody (dilution 1:100) followed by secondary anti-rabbit HRP-conjugated antibody (Dako, Carpinteria, CA, USA). The slides were stained using a Dako Liquid DAB^+^ Substrate Chromogen System (Dako) and counterstained using Dako Automation Hematoxylin Histological Staining Reagent (Dako). ASAH1 staining of the TMA slides was scored by Dr. Xuchen Zhang, who was blinded to the slides’ identity. Details on the antibodies used for IHC analyses are listed in Supplementary Table 1.

### 2.6 Metabolomic analysis

A375 cells expressing *ASAH1* or non-specific shRNA were analyzed for alterations in metabolic pathways using the capillary-electrophoresis time-of-flight mass spectrometry-based basic scan profiling method developed by Human Metabolome Technologies (Boston, MA, USA). Briefly, the cells were incubated in duplicate, and 1 × 10^6^ cells were analyzed for each condition. Samples were prepared according to the recommendations of Human Metabolome Technologies. For data analysis, peaks detected during spectrometric analysis were extracted using MasterHands version 2.17.1.11 automated integration software (developed at Keio University, Tokyo, Japan) to determine mass/charge ratio (*m*/*z*), migration time, and peak area. The peak area was converted into the relative peak area using the following equation: relative peak area = metabolite peak area/internal-standard peak area × number of cells. The peak detection limit was determined based on a signal-to-noise ratio of 3. Putative metabolites were assigned based on the *m*/*z* and migration time using Human Metabolomic Technologies’ standard and known-unknown peak libraries. All the metabolite concentrations were calculated by normalizing the peak area of each metabolite to the area of the internal standard and by comparing with standard curves obtained from a 100 μM single-point calibration. The peak profile of putative metabolites was represented on metabolic pathway maps using the Visualization and Analysis of Networks containing Experimental Data (VANTED) software (http://vanted.ipk-gatersleben.de).

### 2.7 Measurement of cellular pipecolic acid levels

Pipecolic acid measurement was performed as previously described [24]. Briefly, cell extracts were prepared in 95% ethanol. To remove lysine, the cell extracts were passed via gravity through Bio-Rex 70 cation-exchange resin (Bio-Rad) pre-equilibrated at a pH of 4.7 and washed with deionized water. The eluate was concentrated, dried using evaporation, mixed with a nitrous acid solution, and left to react in a glycerol bath at 122 °C for 5 min. The reaction mixture was dried, dissolved in 500 μl of water, and desalted using AG 50W-X8 and AG 1-X8 columns (Bio-Rad). The eluate was dried again and solubilized in 500 μl of water. The samples were transferred into fresh tubes and dried via evaporation at 80–90 °C under ventilation. Ninhydrin solution (3% w/v in a 9:1 n-butanol:citrate buffer pH 4.2 mixture) was added. The tubes were then heated in boiling water for 3 min and cooled rapidly in water. The solution was diluted in 200 μl of ethyl acetate, and the absorbance of the solution at 580 nm was immediately measured.

### 2.8 Confocal microscopy

#### 2.8.1 PMP70 staining

Melanoma cells (10 × 10^3^) expressing either *ASAH1* or non-specific shRNA were plated in multi-well chambered slides. After 24 h, the cells were washed with PBS and fixed with 3.7% paraformaldehyde. The cells were permeabilized using 0.3% Triton X-100. After washing with PBS, the slides were blocked using 5% BSA in PBS. The cells were then probed with PMP70 primary antibody (1:200) (see Supplementary Table 1). After washing, the cells were incubated with HRP-conjugated secondary AlexaFluor-488 anti-rabbit antibody (1:1000) (see Supplementary Table 1). Fluorescence images were collected using a LEICA SP5 confocal laser scanning microscope.

### 2.9 Measurement of total cellular ROS

Melanoma cells (5 × 10^4^) expressing either *ASAH1* or non-specific shRNA were labeled with 2′,7′-dichlorofluorescin diacetate (H2DCFDA, Invitrogen) according to the manufacturer's protocol. ROS levels were measured using fluorescence-activated cell sorting on a FACSCalibur (Becton Dickinson, Franklin Lakes, NJ, USA). Data from 10,000 cells were analyzed using FlowJo software.

### 2.10 Peroxisomal ROS measurement

To directly measure the peroxisomal ROS level, we used the GFP-based reporter roGFP2 [25] fused with the peroxisomal serine-lysine-leucine (SKL) targeting sequence. Melanoma cells were transfected with vector encoding roGFP2 for 48 h and fixed in 3.7% paraformaldehyde. The peroxisomal redox status was analyzed using confocal microscopy. Images of peroxisomes were collected using two different excitation/emission settings: excitation 410 nm/emission 528 nm (ex410/em528) and excitation 488 nm/emission 528 nm (ex488/em528). roGFP2 is sensitive to redox conditions, with a larger 410 nm/488 nm fluorescence intensity ratio indicating increased oxidative stress [[26], [27], [28], [29], [30]]. Therefore, fluorescent intensities were analyzed using ImageJ, and the 410 nm/488 nm fluorescence intensity ratio was calculated. The fluorescence intensity ratios were normalized to that of cells transfected with non-specific shRNA.

### 2.11 Measurement of cellular ceramide levels

Cellular ceramides were measured in melanoma cells using the ceramide kinase method as previously described [31]. Briefly, ceramide species were extracted in the organic phase of a 1:1:0.9 methanol:chloroform:water mixture and resuspended in micelles. Recombinant ceramide kinase (25 units) was added to the samples, and reactions were initiated by adding 10 μl of [γ-^32^P]ATP (5 μCi, 10 mM in 100 mM of MgCl_2_). After 20 min at 30 °C, the reactions were stopped and lipids were extracted by adding 1.2 ml of 1:1 (v/v) chloroform/methanol. After vortexing, 500 μl of 1 M KCl in 20 mM of MOPS (pH 7.2) was added, and phases were separated using centrifugation. The organic phase was re-extracted three times with 1 M of KCl to reduce the background, and the radioactivity was measured using a scintillation counter (Beckman Coulter).

### 2.12 Mouse tumorigenesis experiments

All of the animal experiments were approved by the Institutional Animal Care and Use Committee (IACUC) at Yale University and the University of Alabama at Birmingham and performed in accordance with the IACUC guidelines.

#### 2.12.1 Mouse tumorigenesis experiments using cells expressing *ASAH1* shRNAs

Athymic nude (NU/J) mice (Stock No. 002019, Jackson Laboratory) aged 4–5 weeks were injected subcutaneously with 5 × 10^6^ melanoma cells expressing *ASAH1* shRNAs or non-specific shRNA. Tumor volume was measured every 3 days and calculated using the formula: length × width^2^ × 0.5.

#### 2.12.2 Mouse tumorigenesis experiments with carmofur and/or vemurafenib treatment

Athymic nude (NU/J) mice (Stock No. 002019, Jackson Laboratory) were injected subcutaneously with 5 × 10^6^ melanoma cells. Vehicle (0.5% methylcellulose), carmofur (80 mg/kg of body weight), or carmofur and vemurafenib (10 mg/kg of body weight) was administered by oral gavage every third day starting the day after the injection of cells until the end of the experiment. Tumor volume was measured every 3 days and calculated using the formula: length × width^2^ × 0.5.

#### 2.12.3 Tail vein injection of cells expressing *ASAH1* shRNAs

A375-MA2 cells stably expressing firefly luciferase under a CMV promoter were generated by co-transfecting the transposon vector piggyBac GFP-Luc and helper plasmid Act-PBase as previously described [32]. Cells with stable transposon integration were selected using blasticidin S (Invitrogen). A375-MA2-GFP-*F-Luc* cells (2.5 × 10^5^) expressing *ASAH1* shRNAs or non-specific shRNA were injected into NSG mice (Stock No. 005557, Jackson Laboratory) via the tail vein. The mice were imaged using an IVIS Spectrum In Vivo Imaging System (PerkinElmer) every week until the end of the experiment. Total luminescence counts of tumor-bearing areas were measured using Living Image in vivo imaging software (PerkinElmer).

### 2.13 Statistical analysis

All the experiments were conducted in at least three biological replicates. Results of individual experiments were expressed as mean ± SEM. For tumor progression in the mice and MTT assays, the statistical analysis was performed by analyzing the area under curve (AUC) using GraphPad Prism version 7.0 for Macintosh (GraphPad Software, San Diego, CA, USA, www.graphpad.com). The p values for rest of the experiments were calculated using the two-tailed unpaired Student's *t* test in GraphPad Prism version 7.0 for Macintosh (GraphPad Software).

## 3 Results

### 3.1 ASAH1 was overexpressed in melanoma

**Figure 1:**
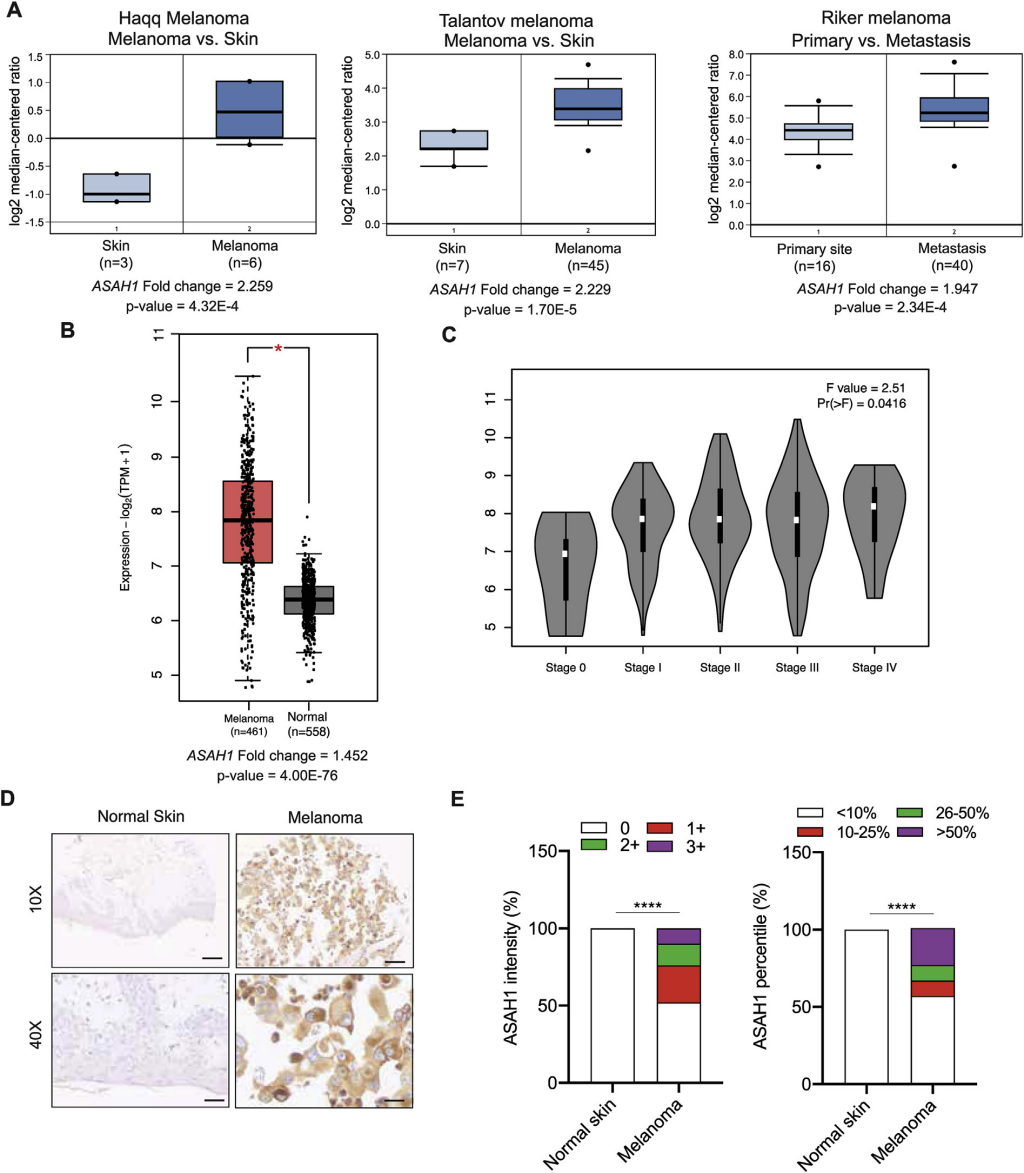
**ASAH1 was overexpressed in melanoma and its inhibition blocked tumor growth.** (A) The relative *ASAH1* mRNA expression in indicated melanoma datasets compared with normal skin vs primary melanoma or primary melanoma vs metastatic melanoma. (B). *ASAH1* mRNA expression is plotted in the Cancer Genome Atlas (TCGA) melanoma samples compared to GTEx and TCGA normal samples combined using Gene Expression Profiling Interactive Analysis (GEPIA). (C) *ASAH1* mRNA expression is plotted in TCGA melanoma samples at indicated stages of melanoma using GEPIA. (D) A tissue microarray (ME2081) of normal skin (n = 16) and melanoma samples (n = 84) was analyzed for the ASAH1 protein expression. Representative images of ASAH1 immunohistochemical staining in normal skin or melanoma samples at 10 × and 40 × magnifications are shown. Scale bar, 100 μm for 10 × and 25 μm for 40 × . (E) Analysis of immunohistochemical data from a TMA with normal skin and melanoma samples. (Left) Normal skin and melanoma samples were scored 0, +1, +2, or +3 based on the ASAH1 staining intensity. A comparison of the average densities of ASAH1 staining in normal skin and melanoma samples is shown. (Right) Normal skin and melanoma samples were scored < 10%, 10–25%, 26–50%, or > 50% based on the ASAH1 staining percentile. A comparison of the average percentiles of ASAH1-expressing cells in normal skin and melanoma samples is shown. Contingency analysis using the Chi-square test was used to determine the significant difference in the ASAH1 expression between normal skin and melanoma samples. Data are presented as mean ± SEM; ∗∗∗∗ represents p values < 0.0001.

When comparing previously published gene expression profiles of patient-derived melanoma samples with normal skin, we found a significant upregulation of ASAH1 in the melanoma samples, and ASAH1 levels increased as melanoma progressed (Figure 1A,B and Supplementary Fig. 1A) [[33], [34], [35], [36], [37]]. Similarly, when evaluating profiles from a panel of multiple cancer cell lines, ASAH1 expression was significantly higher in the melanoma cell lines (Supplementary Figs. 1B and 1C). We also found that ASAH1 expression increased with tumor progression as observed by increased *ASAH1* mRNA expression levels with increasing melanoma stages (Figure 1C). To validate these results, we also analyzed ASAH1 protein expression in a melanoma tissue microarrays (TMAs) consisting of 121 samples of malignant melanoma and 56 normal skin controls using immunohistochemistry. We found that over 50% of the melanoma samples had higher ASAH1 expression compared with the normal skin samples (Figure 1D,E, Supplementary Figs. 2A–B, and Supplementary Tables 2 and 3). Collectively, these results suggest a potentially important role of ASAH1 in melanoma tumor growth and metastasis.

### 3.2 ASAH1 was necessary for melanoma tumor growth and metastasis

**Figure 2:**
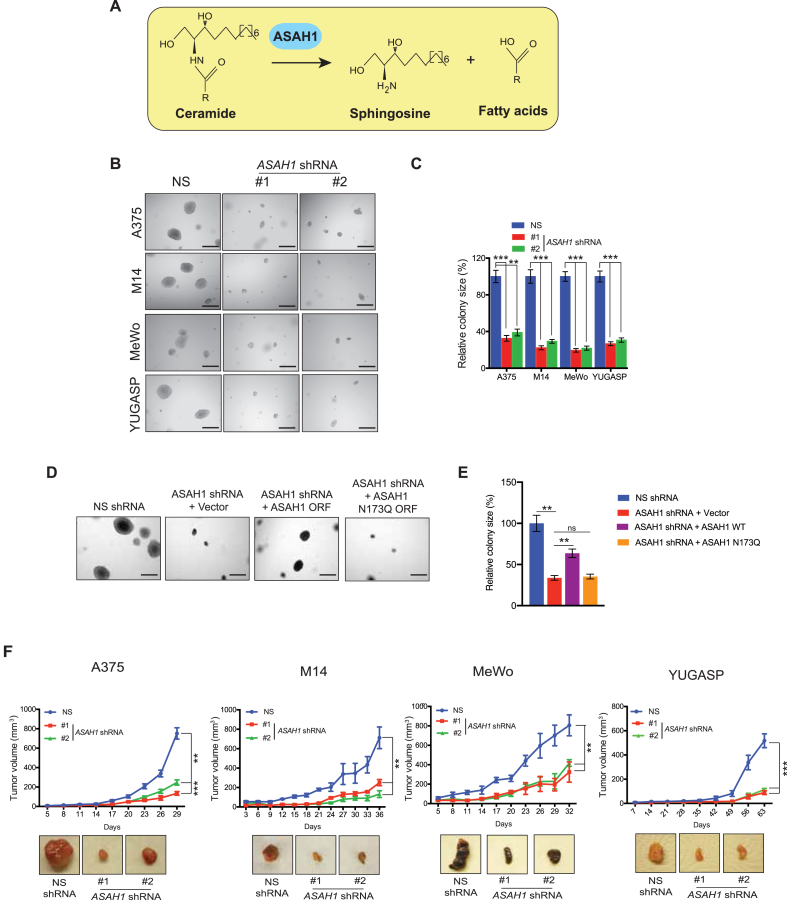
**Loss of ASAH1 blocked melanoma tumor growth.** (A) A schematic showing the enzymatic reaction catalyzed by ASAH1. (B) Representative images of soft-agar colonies for the indicated melanoma cell lines expressing either *ASAH1* shRNAs or NS shRNA. Scale bar, 500 μm. (C) Relative colony size from the soft-agar assay shown in panel B. (D) Representative images of soft-agar colonies for A375 cells expressing NS shRNA alone or *ASAH1* shRNA with empty vector, wild-type ASAH1 vector, or enzymatic activity-defective ASAH1 mutant (ASAH1-N173Q) vector. Scale bar, 500 μm. (E) Relative colony size from the soft-agar assay presented in panel D. (F) The indicated melanoma cell lines expressing either *ASAH1* shRNAs or NS shRNA were subcutaneously injected into the flanks of athymic nude mice. Plots of the average tumor volumes at the indicated times (top) and representative images of tumors (bottom) are shown. Data are presented as mean ± SEM; ∗, ∗∗, ∗∗∗, and ∗∗∗∗ represent p values < 0.05, <0.01, <0.001, and <0.0001, respectively.

ASAH1 catalyzed the hydrolysis of ceramide to generate sphingosine and free fatty acids (Figure 2A). To test whether ASAH1 is necessary for tumor growth, we knocked down *ASAH1* expression in multiple melanoma cell lines (A375, M14, MeWo, and YUGASP) using shRNAs (Supplementary Figs. 3A and 3B) and evaluated anchorage-independent growth using a soft-agar assay. *ASAH1* knockdown significantly reduced the ability of various melanoma cells to form colonies compared with cells expressing non-specific shRNA (Figure 2B,C).

We next investigated whether the acid ceramidase activity of ASAH1 is required for melanoma tumor growth. We generated an ASAH1 mutant ASAH1 N173Q that lacked acid ceramidase activity [38]. We then compared the ability of *ASAH1* N173Q mutant to rescue melanoma growth with that of wild-type *ASAH1* using the soft-agar assay. shRNA-resistant wild-type *ASAH1* rescued tumor growth in the melanoma cell line expressing *ASAH1* shRNA, whereas the shRNA-resistant enzymatically defective *ASAH1* mutant did not (Figure 2D,E and Supplementary Fig. 3C). These results demonstrate that the acid ceramidase activity of ASAH1 is necessary to promote melanoma growth.

To further verify the role of ASAH1 in melanoma tumor growth, we performed in vivo experiments. We injected various melanoma cell lines (A375, M14, MeWo, and YUGASP) expressing either *ASAH1* shRNAs or a non-specific shRNA subcutaneously into the flanks of the athymic nude mice and monitored melanoma tumor growth. Consistent with our soft-agar results, knockdown of *ASAH1* significantly inhibited melanoma tumor growth compared with non-specific shRNA in vivo (Figure 2F).

**Figure 3:**
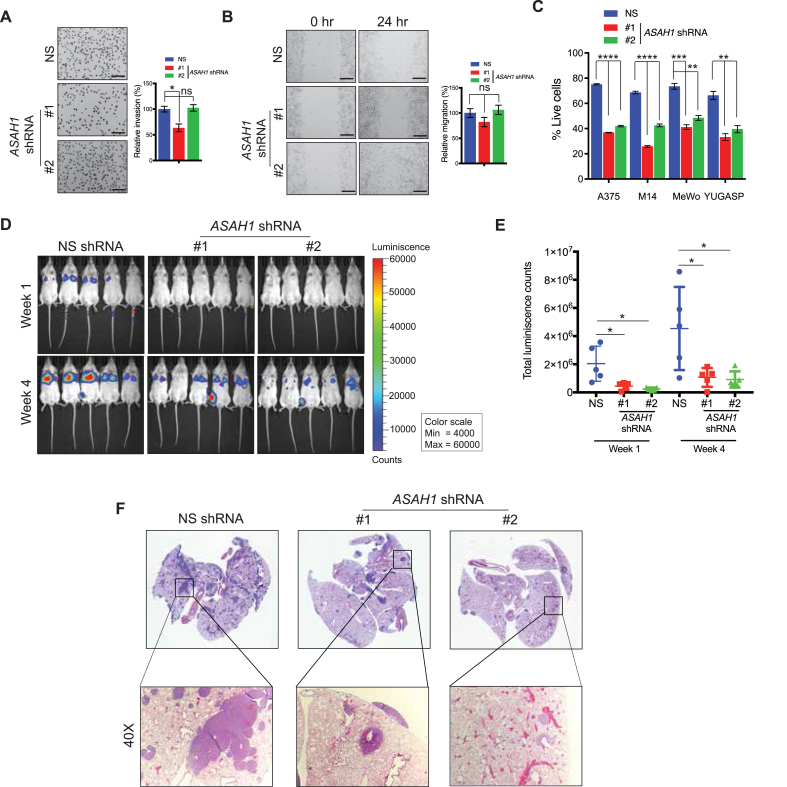
**ASAH1 inhibition induced anoikis and inhibited melanoma growth in lungs.** (A) A375 cells expressing *ASAH1* shRNAs or non-specific (NS) shRNA were analyzed using a Matrigel invasion assay. (Left) Representative images of invasion are shown. (Right) Relative invasion (%) is shown. Scale bar, 200 μm. (B) A375 cells expressing *ASAH1* shRNAs or NS shRNA were analyzed using a wound-healing assay. (Left) Representative images at the indicated times are shown. (Right) Relative migration (%) is shown. Scale bar, 200 μm. (C) Indicated melanoma cell lines expressing *ASAH1* shRNAs or NS shRNA were plated on ultra-low attachment plates, and survival was measured at day 7. The relative percent of surviving cells at day 7 is shown. (D) A375-MA2-*F-Luc* cells expressing *ASAH1* shRNAs or NS shRNA were injected via tail veins of NSG mice to mirror metastatic growth in lungs. Bioluminescence images of the mice from the indicated groups at weeks 1 and 4 are shown. (E) Quantitation of the imaging data presented in panel D. (F) Representative images of H&E-stained lung sections with 40 × images of inset regions showing the histology of tumor metastases. Data are presented as mean ± SEM; ∗, ∗∗, ∗∗∗, and ∗∗∗∗ represent p values < 0.05, <0.01, <0.001, and <0.0001, respectively; ns represents not significant p values.

Additionally, we assessed whether *ASAH1* knockdown affects melanoma metastasis. Therefore, we analyzed the effects of *ASAH1* knockdown on metastatic cell migration, invasion, and anoikis. Knockdown of *ASAH1* did not significantly affect the invasiveness or migration of melanoma cells compared with cells expressing non-specific shRNA (Figure 3A,B) but did induce anoikis (Figure 3C). Anoikis resistance, a key feature of transformed and metastatic cells, promotes cell growth following detachment from the extracellular matrix and the survival of circulating tumor cells [39]. Therefore, we investigated if *ASAH1* expression plays a role in melanoma metastasis in vivo. We administered melanoma cells expressing *ASAH1* or non-specific shRNA via the tail vein in NOD-SCID gamma (NSG) mice. In tail vein-based experiments mirroring lung colonization, *ASAH1* knockdown significantly decreased the metastatic growth of melanoma cells in the lungs compared with cells expressing non-specific shRNA (Figure 3D–F). Collectively, these results demonstrate that genetic inhibition of ASAH1 attenuates melanoma tumor growth and metastatic growth in lungs.

### 3.3 ASAH1 was transcriptionally upregulated through the action of E2F1 in a MAPK pathway-dependent manner

**Figure 4:**
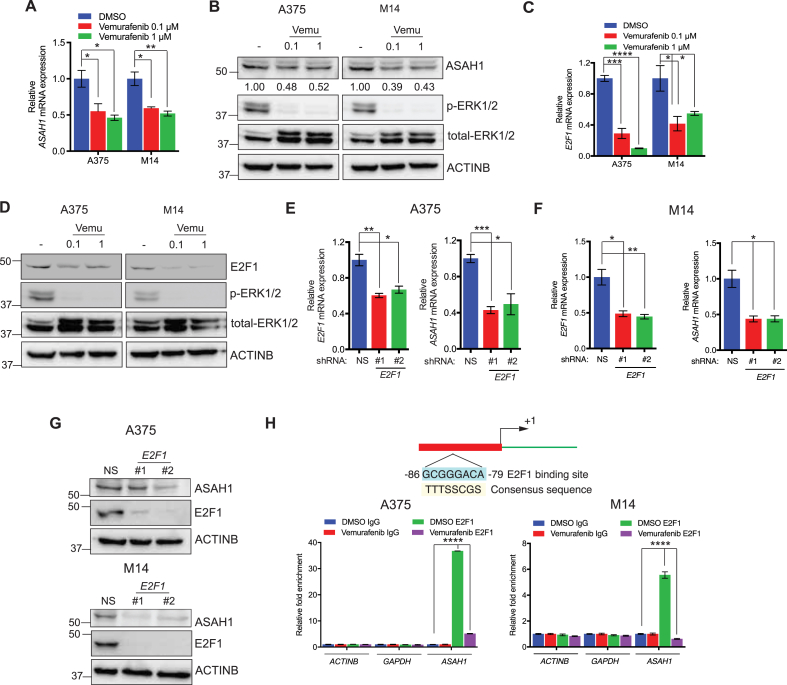
**ASAH1 was transcriptionally upregulated through the action of E2F1 in a MAPK pathway-dependent manner.** (A) Relative *ASAH1* mRNA expression in indicated melanoma cell lines treated with DMSO (−) or vemurafenib (V) (0.1 μM or 1 μM) for 24 h is plotted relative to melanoma cell lines treated with DMSO. (B) The indicated melanoma cell lines treated with DMSO (−) or vemurafenib (0.1 μM or 1 μM) for 24 h were analyzed for the indicated proteins using immunoblotting. ACTINB was used as a loading control. (C) *E2F1* mRNA expression in the indicated melanoma cell lines treated with DMSO (−) or vemurafenib (0.1 μM or 1 μM) for 24 h relative to melanoma cell lines treated with DMSO. (D) The indicated melanoma cell lines were treated with DMSO (−) or vemurafenib (0.1 μM or 1 μM) for 24 h. The expression of the indicated proteins was measured using immunoblotting. ACTINB was used as a loading control. (E) A375 cells expressing *E2F1* shRNAs or non-specific (NS) shRNA were analyzed for *E2F1* and *ASAH1* mRNA expression. *E2F1* and *ASAH1* mRNA expression in *E2F1* shRNA expressing cells was plotted relative to NS shRNA-expressing cells. (F) M14 cells expressing either *E2F1* shRNAs or NS shRNA were analyzed for *E2F1* and *ASAH1* mRNA expression. *E2F1* and *ASAH1* mRNA expression in *E2F1* shRNA-expressing cells was plotted relative to NS-shRNA-expressing cells. (G) The expression of the indicated proteins was analyzed in A375 and M14 cells expressing either *E2F1* shRNAs or NS shRNA by immunoblotting. ACTINB was used as a loading control. (H) A375 and M14 cells were treated with DMSO or vemurafenib (1 μM) for 24 h and analyzed for E2F1 recruitment to either the *ASAH1* promoter or *ACTINB* and *GAPDH* control promoters using a chromatin immunoprecipitation assay. IgG was used as a negative control. Fold-enrichment relative to IgG is shown. The coordinates of E2F1-binding sites on the *ASAH1* promoter are shown in the top panel. Data are presented as mean ± SEM; ∗, ∗∗, ∗∗∗, and ∗∗∗∗ represent p values < 0.05, <0.01, <0.001, and <0.0001, respectively.

We next determined the mechanism leading to ASAH1 overexpression in melanoma. The MAPK pathway is constitutively active in a large percentage of melanomas because of activating *NRAS* and *BRAF* mutations or inactivating *NF1* mutations [3]. Therefore, we investigated if the MAPK pathway activity is required for transcriptional ASAH1 overexpression in melanoma. We treated the melanoma cell lines A375 and M14 with the BRAF kinase inhibitor vemurafenib. Vemurafenib treatment downregulated the expression of *ASAH1* mRNA (Figure 4A) and protein (Figure 4B) compared with vehicle-treated cells. Collectively, these results show that the MAPK pathway transcriptionally upregulates ASAH1 expression in melanoma.

To identify the transcription factors downstream of the MAPK pathway that regulate ASAH1 expression in melanoma cells, we analyzed the sequence of the ASAH1 promoter using the transcription factor/DNA-binding site prediction programs PROMO and rVISTA 2.0. We identified potential binding sites for 32 transcription factors in the ASAH1 promoter sequence (Supplementary Table 4). We hypothesized that the relevant transcription factor would be regulated by the MAPK pathway similar to ASAH1. Therefore, we treated the melanoma cell lines A375 and M14 with vemurafenib and measured the expression of all 32 transcription factors (Supplementary Fig. 4). E2F1 was the only transcription factor that was downregulated after vemurafenib treatment in both melanoma cell lines (Figure 4C,D and Supplementary Fig. 4).

To determine whether E2F1 regulates ASAH1 transcription, we knocked down *E2F1* expression in the melanoma cell lines A375 and M14. Knockdown of *E2F1* significantly reduced *ASAH1* mRNA and protein levels compared with cells transfected with non-specific shRNA (Figure 4E–G). To determine if E2F1 directly regulates *ASAH1* transcription, we used a chromatin immunoprecipitation (ChIP) assay to investigate the association between E2F1 and the *ASAH1* promoter. The ChIP results confirmed that E2F1 binds the *ASAH1* promoter, and this binding decreased when cells were treated with vemurafenib (Figure 4H). Collectively, these results show that the MAPK pathway increases E2F1 expression, which stimulates *ASAH1* transcription in melanoma cells.

### 3.4 ASAH1 loss promoted peroxisome biogenesis via a ceramide-induced increase in peroxisome proliferator-activated receptor gamma (PPARγ) activity

**Figure 5:**
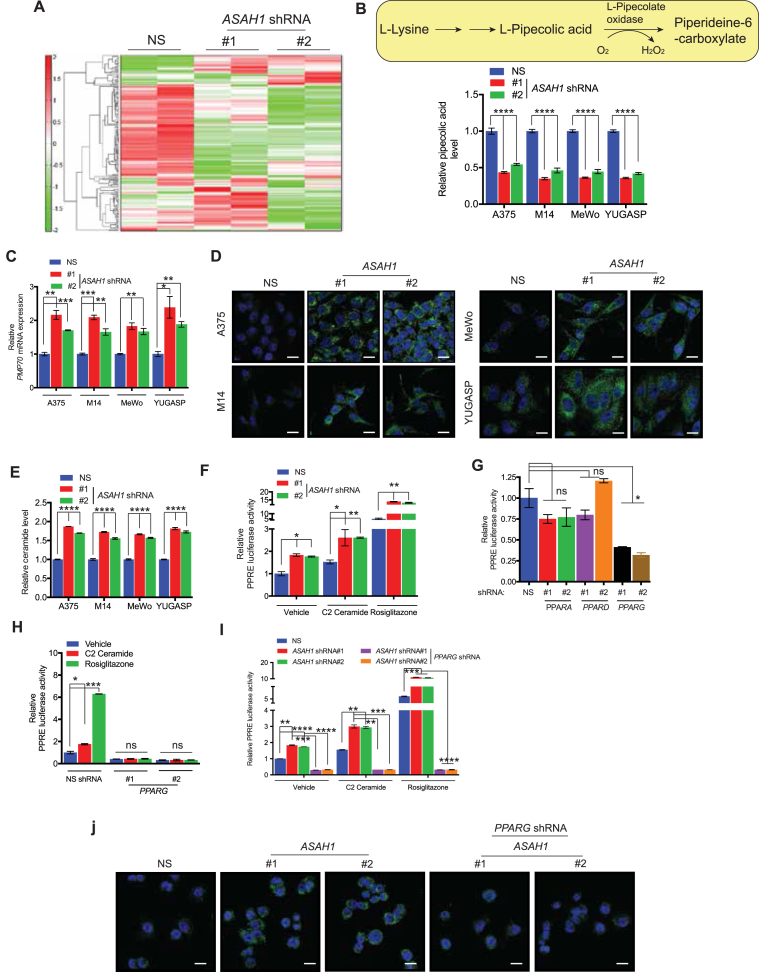
**ASAH1 loss promoted peroxisome biogenesis via a ceramide-induced increase in PPARγ activity.** (A) A heat map showing the metabolite profile of A375 cells expressing *ASAH1* shRNAs or a non-specific (NS) shRNA. (B) (Top) A schematic showing the enzymatic steps leading to generation of pipecolic acid. (Bottom) The relative concentration of pipecolic acid in melanoma cell lines expressing *ASAH1* shRNAs or NS shRNA. (C) The indicated melanoma cell lines expressing *ASAH1* shRNAs or NS shRNA were analyzed for *PMP7*0 mRNA expression. *PMP7*0 mRNA expression is presented relative to melanoma cells expressing NS shRNA. (D) The indicated melanoma cell lines expressing *ASAH1* shRNAs or NS shRNA were analyzed for peroxisome content using PMP70 immunofluorescence and confocal microscopy. Representative images are shown. Scale bar, 20 μm. (E) The indicated melanoma cell lines expressing *ASAH1* shRNAs or NS shRNA were analyzed for total cellular ceramide levels. Ceramide levels in cells expressing *ASAH1* shRNAs are presented relative to that of cells expressing NS shRNA. (F) A375 cells expressing *ASAH1* shRNAs or a NS shRNA were transfected with a PPAR-responsive firefly-luciferase-promoter reporter construct and treated with C2 ceramide (25 μM) or rosiglitazone (25 μM) for 24 h. The cells were then analyzed for luciferase activity. Renilla-luciferase activity was used to normalize the difference in transfection efficiencies. The luciferase activity was plotted relative to A375 cells expressing NS shRNA. (G) A375 cells expressing shRNAs for the indicated PPAR isoforms were transfected with PPRE-responsive firefly-luciferase-promoter reporter construct and analyzed for luciferase activity. Renilla-luciferase activity was used to normalize the difference in transfection efficiencies. Luciferase activity was plotted relative to A375 cells expressing NS shRNA. (H) A375 cells expressing *PPARG* shRNAs or NS shRNA were transfected with PPRE-responsive firefly-luciferase promoter construct, treated with C2 ceramide (25 μM) or rosiglitazone (25 μM) for 24 h, and analyzed for luciferase activity. Renilla-luciferase activity was used to normalize the difference in transfection efficiencies. Luciferase activity was plotted relative to A375 cells expressing NS shRNA. (I) A375 cells expressing NS shRNA alone, *ASAH1* shRNAs alone, or *ASAH1* shRNAs with *PPARG* shRNA were analyzed for PPRE-driven firefly-luciferase activity. Normalized luciferase activity was plotted relative to A375 cells expressing NS shRNA. (J) A375 cells expressing NS shRNA alone, *ASAH1* shRNAs alone, or *ASAH1* shRNAs with *PPARG* shRNA were analyzed for peroxisome content using PMP70 immunofluorescence and confocal microscopy. Representative immunofluorescence images are shown. Scale bar, 20 μm. Data are presented as mean ± SEM; ∗, ∗∗, ∗∗∗, and ∗∗∗∗ represent p values < 0.05, <0.01, <0.001, and <0.0001, respectively.

Once we established the role of ASAH1 in melanoma tumor growth and metastasis and the mechanism driving its overexpression, we investigated the mechanism by which ASAH1 promotes tumor growth. Because ASAH1 is a metabolic enzyme, we used a global metabolomic analysis to understand its effect on downstream metabolic targets. The metabolites of A375 cells expressing non-specific shRNA or *ASAH1* shRNAs were analyzed using capillary-electrophoresis time-of-flight mass spectrometry in two modes: one for cationic and one for anionic metabolites. We detected 223 different metabolites in different metabolic pathways (Figure 5A and Supplementary Table 5). Notably, pipecolic acid levels were reduced in the cells expressing *ASAH1* shRNAs compared with those expressing non-specific shRNA (Supplementary Table 5). Based on the results of the metabolomic analysis, we used a secondary validation assay to monitor changes in pipecolic acid after knocking down *ASAH1*. In complete agreement with the results of the metabolomic analysis, knockdown of *ASAH1* in melanoma cell lines (A375, M14, MeWo, and YUGASP) resulted in reduced pipecolic acid levels (Figure 5B). Pipecolic acid is oxidized in peroxisomes by l-pipecolate oxidase [40,41]. Therefore, reduced pipecolic acid levels indicated that *ASAH1* knockdown potentially increased peroxisomal activity. Based on these results, we investigated whether *ASAH1* knockdown increased peroxisome biogenesis. Therefore, we measured PMP70 levels. PMP70 is a peroxisome marker used to quantify the number of peroxisomes in cells [42]. *ASAH1* knockdown in melanoma cell lines (A375, M14, MeWo, and YUGASP) increased PMP70 mRNA levels compared with cells expressing non-specific shRNA (Figure 5C). To verify these results, we incubated cells with PMP70 antibody to detect the total peroxisomal content and performed confocal microscopy. This experiment confirmed that PMP70 expression increased (Figure 5D). Similar increases in PMP70 and ceramide levels and decreases in pipecolic acid levels were also observed in tumors derived from *ASAH1* shRNA expressing A375 and M14 melanoma xenografts (Supplementary Figs. 5A–C). However, no significant increase in the plasma levels of ceramide was observed in these mice (Supplementary Fig. 5D). Collectively, these results show that *ASAH1* knockdown increases peroxisome biogenesis.

Ceramide can act as a ligand for and activator of PPARs [43]. Therefore, we investigated if knockdown of *ASAH1* increased ceramide levels. As expected, *ASAH1* knockdown increased ceramide levels in melanoma cells (Figure 5E). We then measured PPAR activity using a PPAR-responsive element (PPRE)-driven firefly-luciferase reporter [44] in melanoma cells expressing *ASAH1* or non-specific shRNA. We found that melanoma cells expressing *ASAH1* shRNAs showed significantly higher firefly-luciferase reporter activity than cells expressing non-specific shRNA (Figure 5F). We then investigated if the increased PPAR activity was due to the *ASAH1* knockdown-induced increase in ceramide levels. We treated melanoma cells expressing *ASAH1* shRNAs or non-specific shRNA with C2 ceramide, a cell-permeable ceramide analog, to mimic the ceramide levels seen with *ASAH1* knockdown. Treatment of melanoma cells expressing non-specific shRNA with C2 ceramide increased PPAR-responsive firefly-luciferase reporter activity compared with vehicle-treated cells expressing non-specific shRNA (Figure 5F). Similarly, treatment of melanoma cells expressing non-specific shRNA with rosiglitazone, a PPARγ agonist [45], also increased PPAR-responsive firefly-luciferase reporter activity compared with vehicle-treated cells expressing non-specific shRNA (Figure 5F). Furthermore, rosiglitazone-stimulated PPAR-responsive firefly-luciferase reporter activity was higher in melanoma cells expressing *ASAH1* shRNAs compared with those expressing non-specific shRNA (Figure 5F).

PPARs are a family of nuclear transcription factors that regulate lipid metabolism in response to various lipid agonists. They act by binding peroxisome proliferator-responsive element [[46], [47], [48]]. In humans, there are three PPAR genes, *α*, *γ*, and *δ*, with varying tissue distributions as well as common and distinct lipid agonists [49]. Therefore, we investigated which PPAR isoforms were involved in the increased peroxisome biogenesis following *ASAH1* knockdown. We knocked down the expression of *PPARα*, *γ*, and *δ* using shRNAs (Supplementary Figs. 6A–6B). Knockdown of *PPARγ* significantly decreased PPAR-responsive firefly-luciferase reporter activity compared with cells expressing non-specific shRNA (Figure 5G). Knockdown of *PPARγ* also strongly inhibited C2 ceramide- and rosiglitazone-induced PPAR-responsive firefly-luciferase reporter activity (Figure 5H).

To confirm the involvement of *PPARγ* in melanoma growth inhibition following ASAH1 loss, we simultaneously knocked down *PPARγ* and *ASAH1* in melanoma cells. Simultaneous knockdown of PPAR*γ* and *ASAH1* inhibited the ability of C2 ceramide or rosiglitazone to activate PPAR-responsive firefly-luciferase reporter activity in melanoma cells (Figure 5I). PMP70 levels were also reduced upon double knockdown of *PPARγ* and *ASAH1* compared with *ASAH1* knockdown alone (Figure 5J). Additionally, anchorage-independent growth was significantly rescued in A375 cells with a double knockdown of *PPARγ* and *ASAH1* (Supplementary Figs. 6C–D). Collectively, these results demonstrate that ASAH1 loss increases ceramide levels, which activates *PPARγ-*induced peroxisome biogenesis.

### 3.5 ASAH1 regulated melanoma growth in a peroxisomal ROS-dependent manner

**Figure 6:**
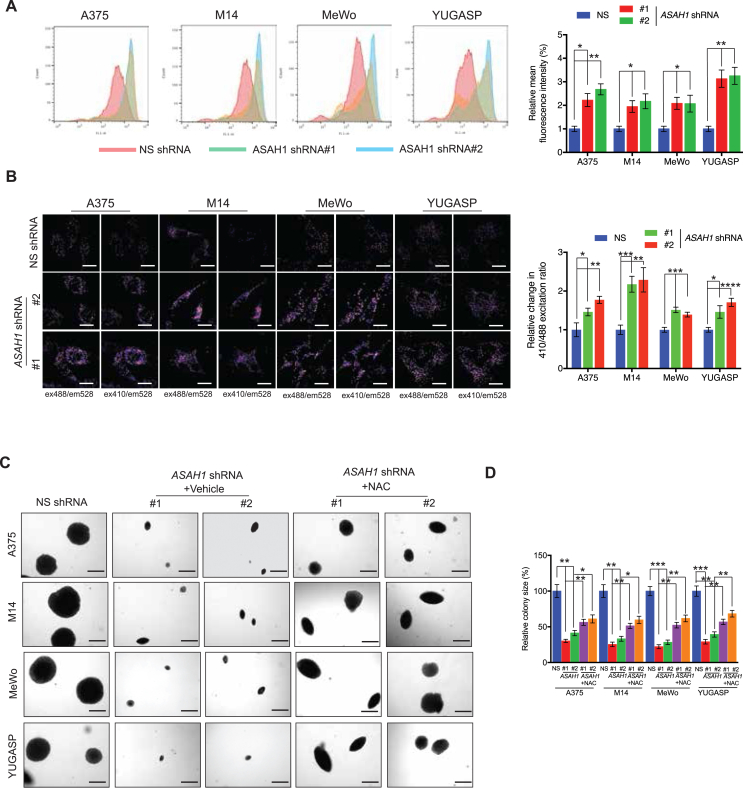
**ASAH1 loss increased ROS production due to increased peroxisomal biogenesis.** (A) The indicated melanoma cell lines expressing NS shRNA or *ASAH1* shRNAs were stained with DCF-DA and analyzed for ROS using FACS analysis. Representative histograms are shown (left), and mean fluorescence intensities (MFI) are plotted as a bar diagram (right). (B) The indicated melanoma cell lines expressing NS shRNA or *ASAH1* shRNAs were analyzed for peroxisomal redox status using roGFP2 via confocal microscopy. The images of peroxisomes (left) were collected using two different excitation/emission settings: excitation 410 nm/emission 528 nm (ex410/em528) and excitation 488 nm/emission 528 nm (ex488/em528). Emission fluorescence intensities at 528 nm were analyzed using ImageJ, and the intensity ratios between ex410/em528 and ex488/em528 were calculated. The intensity ratios were normalized to the ratio of cells transfected with NS shRNA (right). Representative immunofluorescence images are shown. Scale bar: 20 μm. (C) The indicated melanoma cell lines expressing NS shRNA or *ASAH1* shRNAs were treated with DMSO or N-acetylcysteine (NAC, 10 mM) every three days during the soft-agar assay. Representative images of the soft-agar plates are shown. Scale bar, 500 μm. (D) Relative colony size from the soft-agar assay presented in panel C. Data are presented as mean ± SEM; ∗, ∗∗, ∗∗∗, and ∗∗∗∗ represent p values < 0.05, <0.01, <0.001, and <0.0001, respectively.

Peroxisomes regulate cellular reactive oxygen species (ROS) levels [50]. Therefore, we measured ROS levels following *ASAH1* knockdown using a dichloro-dihydro-fluorescein diacetate (DCF-DA)-based assay. ROS levels were higher in melanoma cells expressing *ASAH1* shRNA than those expressing non-specific shRNA (Figure 6A). We then measured peroxisome-specific ROS production using a peroxisome-targeted roGFP2 construct, which acts as peroxisomal redox-sensitive probe [25]. *ASAH1* knockdown in melanoma cells increased peroxisomal ROS levels compared with cells expressing non-specific shRNA (Figure 6B).

Furthermore, to test whether the increased ROS production resulting from *ASAH1* knockdown contributes to the inhibition of melanoma growth, we treated cells with antioxidant N-acetylcysteine (NAC) and performed soft-agar assays. Treatment of *ASAH1*-knockdown melanoma cells with NAC rescued melanoma growth in soft agar (Figure 6C,D), demonstrating that the *ASAH1* knockdown-induced increase in peroxisomal ROS plays a role in inhibiting melanoma growth. Taken together, these data show that ASAH1 knockdown results in increased peroxisome biogenesis and peroxisome-derived ROS production, which suppresses melanoma growth.

### 3.6 Pharmacological inhibition of ASAH1 attenuated melanoma growth and increased responses to drugs targeting melanoma

**Figure 7:**
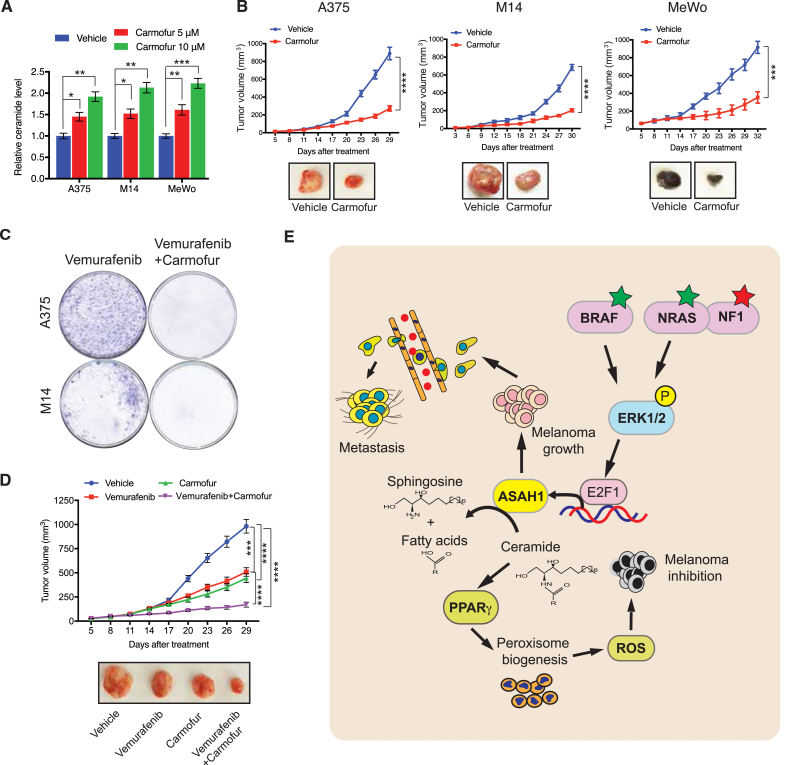
**Pharmacological regulation of ASAH1 inhibited melanoma growth and increased response to drugs targeting melanoma.** (A) The indicated melanoma cell lines were analyzed for total cellular ceramide levels. The relative ceramide levels following treatment with DMSO or carmofur (5 or 10 μM) for 24 h are shown. (B) The indicated melanoma lines were injected subcutaneously into the flanks of athymic nude mice (n = 5). After 1 week, the mice were treated orally with vehicle (0.5% methyl cellulose) or carmofur (80 mg/kg). The average tumor volumes at the indicated timepoints are plotted (top), and representative tumor images are shown (bottom). (C) The indicated melanoma cell lines were treated with 2 μM of vemurafenib alone or in combination with 5 μM of carmofur for four weeks. Images of representative plates with surviving colonies are shown. (D) A375 cells were subcutaneously injected into the flanks of athymic nude mice (n = 5). When the tumor volume reached approximately 50 mm^3^, the mice were orally administered vehicle (0.5% methyl cellulose), carmofur (80 mg/kg), or a combination of carmofur and vemurafenib (10 mg/kg). Average tumor volumes at the indicated time points are plotted (top), and representative tumor images are shown (bottom). (E) A model summarizing the role of ASAH1 in the regulation of peroxisomal ROS via ceramide to promote melanoma growth and metastasis. Data are presented as mean ± SEM; ∗, ∗∗, ∗∗∗, and ∗∗∗∗ represent p values < 0.05, <0.01, <0.001, and <0.0001, respectively.

Because genetic inhibition of ASAH1 inhibited melanoma growth, we investigated whether a pharmacological ASAH1 inhibitor could similarly inhibit melanoma growth. Therefore, we tested carmofur, a small-molecule ASAH1 inhibitor, for its ability to increase ceramide levels [51]. Carmofur treatment of melanoma cell lines increased ceramide levels (Figure 7A) and reduced survival (Supplementary Fig. 7A). C2 ceramide treatment similarly reduced survival of melanoma cell lines (Supplementary Fig. 7B). Based on these results, we tested melanoma tumor growth inhibition using carmofur in a human cell line xenograft-based mouse model of melanoma. We injected melanoma cell lines subcutaneously into the flank of the athymic nude mice and treated them with carmofur. We found that melanoma tumor growth was inhibited in mice treated with carmofur compared with those treated with vehicle (Figure 7B). We also observed increases in levels of PMP70 and ceramide and decreases in pipecolic acid levels in tumor-derived melanoma xenograft-bearing mice treated with carmofur (Supplementary Figs. 8A–C). However, no significant changes were observed in the plasma levels of ceramide in these mice (Supplementary Fig. 8D).

We next evaluated whether ASAH1 inhibition affects the sensitivity of melanoma cells to BRAF kinase inhibitors. First, we tested whether *ASAH1* knockdown alters the sensitivity of BRAF-mutant melanoma cells to the BRAF kinase inhibitor vemurafenib in vitro. *ASAH1*-knockdown cells were significantly more sensitive to vemurafenib treatment than cells expressing non-specific shRNA (Supplementary Fig. 9A). Similarly, *ASAH1*-knockdown cells were more sensitive to C2-ceramide-induced inhibition of cell survival (Supplementary Fig. 9B). Additionally, the combination of carmofur and vemurafenib more potently inhibited melanoma cell survival than carmofur alone (Supplementary Fig. 9C). We also performed a long-term clonogenic assay to recapitulate the emergence of acquired resistance to vemurafenib as it occurs in clinic. The combination of vemurafenib and carmofur prevented the emergence of vemurafenib-resistant clones (Figure 7C). We tested the effectiveness of the vemurafenib and carmofur combination compared with vemurafenib alone for treating BRAF-mutant melanoma in vivo. To this end, we injected the BRAF-mutant melanoma cell line A375 subcutaneously into the flanks of the athymic nude mice and treated them with either carmofur alone, vemurafenib alone, or a combination of carmofur and vemurafenib. Administration of the combination treatment inhibited tumor growth more potently than vemurafenib or carmofur alone (Figure 7D). Collectively, these results demonstrate that the ASAH1-driven ceramide metabolism pathway can be inhibited pharmacologically to treat melanoma and enhance outcomes when combined with other targeted therapeutics, such as BRAF kinase inhibitors.

## 4 Discussion

Cancer cells depend on deregulated metabolic pathways to fulfill their growth requirements and thus these metabolic pathways become cancer cell liabilities for their survival [[13], [14], [15], [16]]. In this study, we identified that ASAH1 is overexpressed in melanoma cells and its loss inhibits melanoma tumor growth and metastasis by suppressing peroxisome biogenesis and inhibiting peroxisome-associated ROS production (Figure 7E).

Oncogenic pathways have been previously shown to activate various metabolic pathways and promote tumor growth and metastasis [16]. Specifically, the oncogenic proteins RAS, MYC, and BRAF have been shown to directly stimulate metabolic pathways by increasing the expression of key metabolic enzymes in cancer cells [16,52]. It has been shown that metabolic alterations downstream of these oncogenes are necessary for their ability to form tumors and in some cases to achieve productive metastasis [16,52]. Ceramide metabolism has been shown to be important for growth and progression of a number of different cancer types [43,53,54]. Ceramide itself can exert tumor-suppressive effects on cancer cells and thus cancer cells develop strategies to metabolize ceramide to non-tumor suppressive metabolites. This in part occurs by activating enzymes that metabolize ceramide [43]. ASAH1 is an acid ceramidase that converts ceramide into sphingosine and free fatty acid [55]. Consistent with its role in metabolizing ceramide, ASAH1 has been shown to be overexpressed in some cancer types [56,57] and predict poor prognosis in gastric cancer and disease progression in breast cancer [22,23]. ASAH1 is necessary for the growth of metastatic prostate cancer, and ASAH1 expression has been observed to be higher in more advanced stages of prostate cancer [20]. Similarly, ASAH1 has been shown to cause drug resistance in acute myeloid leukemia [58] and radio resistance in glioblastoma cells [59].

Our study identified that ASAH1 is a transcriptional target of the MAPK pathway and showed that transcription factor E2F1 was required for MAPK-mediated upregulation of ASAH1. We also showed that melanoma cells depend on the enzymatic activity of ASAH1 and that its expression is necessary for melanoma tumors and metastatic growth in mice.

Ceramide was originally believed to serve only as a structural lipid with no role in signaling pathway regulation. However, recent evidence suggests that ceramides are bioactive lipids that regulate various signaling pathways and can induce apoptosis [43,60]. For example, ceramide has been shown to induce mitochondrial activation and apoptosis via BAX [61]. Additionally, ceramide has been shown to downregulate the expression of cell-surface transporters, such as GLUT1, and to regulate glucose metabolism [62]. Ceramide has also been shown to regulate other nutrient transporters, such as the amino acid transporter, mCAT1, causing a starvation state and inducing apoptosis [62]. Our global metabolomic analysis identified that ASAH1 may have an effect on glucose metabolism (Supplementary Table 5), which was consistent with a previous study [62]. We have also identified a new role for ASAH1 in promoting melanoma growth. When ASAH1 activity is lost or inhibited, ceramide levels increase, which in turn enhances PPARγ activity and promotes peroxisome biogenesis. Peroxisomes along with mitochondria are sites of fatty acid degradation, and peroxisomes specifically metabolize long and very long chain fatty acids [63]. In contrast to mitochondria, FADH2 produced during peroxisomal beta-oxidation is converted into hydrogen peroxide whereas in mitochondria FADH2 is used to generate ATP [64,65]. Mitochondria and peroxisome are metabolically connected, with mitochondria serving as source of many peroxisomal enzymes and cargo [66]. Uncontrolled peroxisome biogenesis increases peroxisomal ROS production. We showed that ROS regulation by ASAH1 through reducing peroxisome biogenesis facilitates melanoma growth, revealing an important role of peroxisomal biogenesis regulation in melanoma growth. These studies also show how metabolic enzymes such as ASAH1 can regulate organelle biogenesis, such as that of peroxisomes, and further highlights the importance of peroxisomes as an important organelle in cancer growth control.

It is important to mention, however, that there seems to be a dual function for ASAH1 by which it promotes cancer growth and progression. The first function is to reduce the tumor suppressive lipid ceramide by reducing its growth inhibitor effects, which will benefit melanoma cells. The second function involves metabolism of ceramide by ASAH1, which produces fatty acid and sphingosine. Sphingosine can then be converted into sphingosine-1-phosphate (S1P). Fatty acid can be used as building blocks on which melanoma cells can grow. S1P signals via G protein-coupled S1P receptors to regulate cell–cell and cell–matrix adhesion and therefore can influence cell migration, differentiation, and survival. In the present study, we monitored the effect of ceramide on peroxisome biogenesis and showed that this is important for mediating the effect of ASAH1 on melanoma growth. Therefore, we believe this aspect of ASAH1 function is critical for its function in promoting melanoma; however, it is also worth noting that we measured the effect of ASAH1 inhibition in our experiments in which ceramide accumulated and showed that it has a tumor-suppressive effect. In this regard, it is important to mention that unlike ASAH1 shRNAs, some of the effects of carmofur can originate in mice due to its in vivo effects on other tissues when it is systemically delivered.

Collectively, our studies demonstrated that the metabolic enzyme ASAH1 promotes melanoma growth through suppressing peroxisome biogenesis and attenuating peroxisome-induced ROS production. Because ASAH1 is an enzyme for which small-molecule inhibitors are available, its inhibition could be a pharmacologically tractable approach for treating melanoma. Using one such inhibitor, carmofur, we also established the feasibility of such an approach. Thus, our study identified a therapeutically targetable metabolic vulnerability of melanoma.

## Author contributions

PM and NW conceived the experiments. PM, RJ, and NW designed the experiments. PM conducted most of the experiments. RJ performed the immunofluorescence experiments. RJ and AN helped with the animal experiments. PM, RJ, AN, and NW analyzed and interpreted the data. XZ analyzed the immunohistochemistry and mouse histology data and provided images. PM and NW prepared the figures and co-wrote the manuscript.
