# Supplementary material for: N-acylsphingosine amidohydrolase 1 promotes melanoma growth and metastasis by suppressing peroxisome biogenesis-induced ROS production
Source: Mol Metab. 2021 Mar 23;48:101217. doi: 10.1016/j.molmet.2021.101217 (PMC8081993; doi:10.1016/j.molmet.2021.101217)
Supplement: Supplementary file 1 — Multimedia component 1 [file mmc1.docx]

**SUPPLEMENTARY INFORMATION**

**SUPPLEMENTARY FIGURE LEGENDS**

**
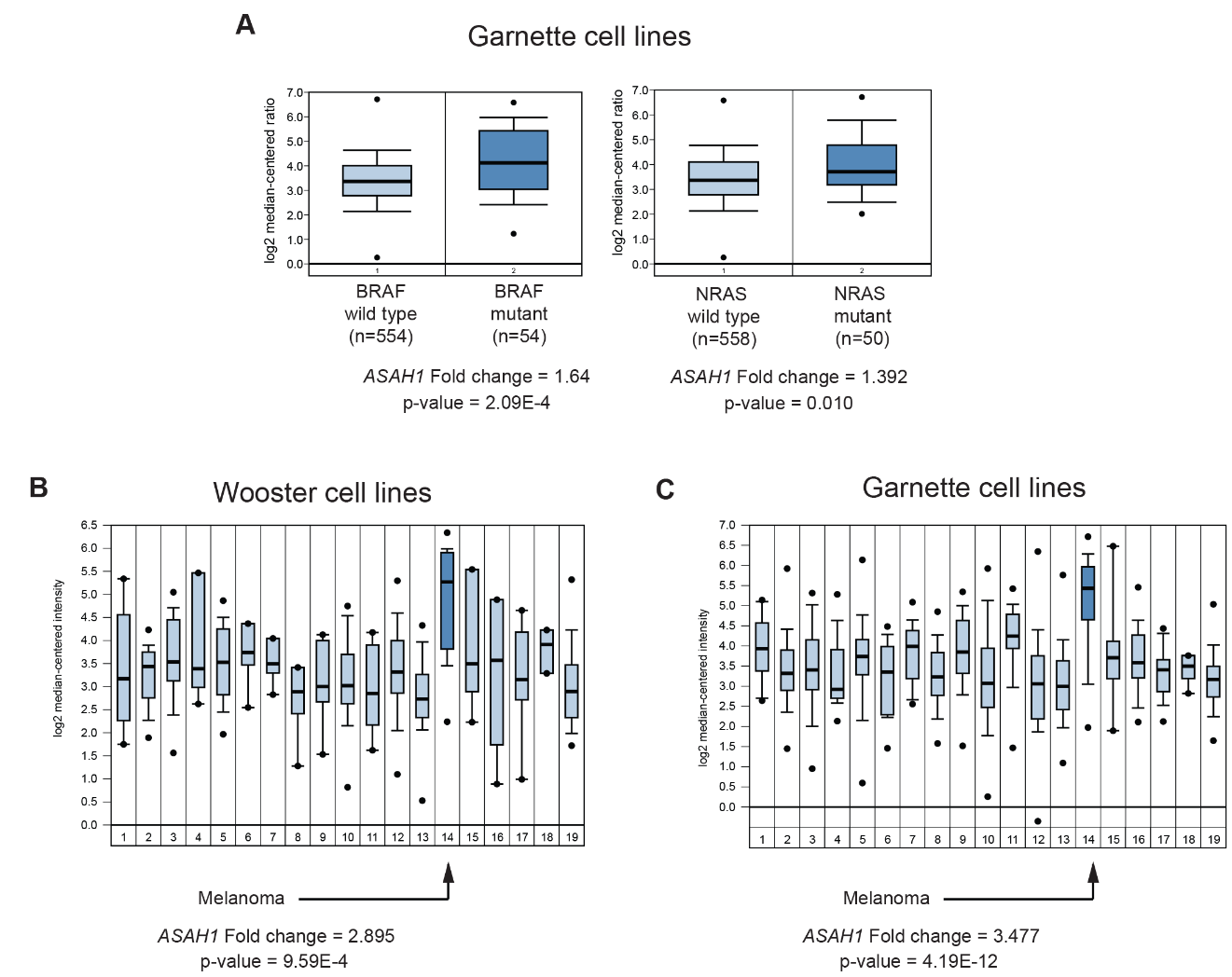
**

**Supplementary Figure 1. ASAH1 is overexpressed in melanoma**. (A-C) The indicated melanoma sample datasets were analyzed for *ASAH1* mRNA expression. The relative *ASAH1* mRNA expression is presented.

**
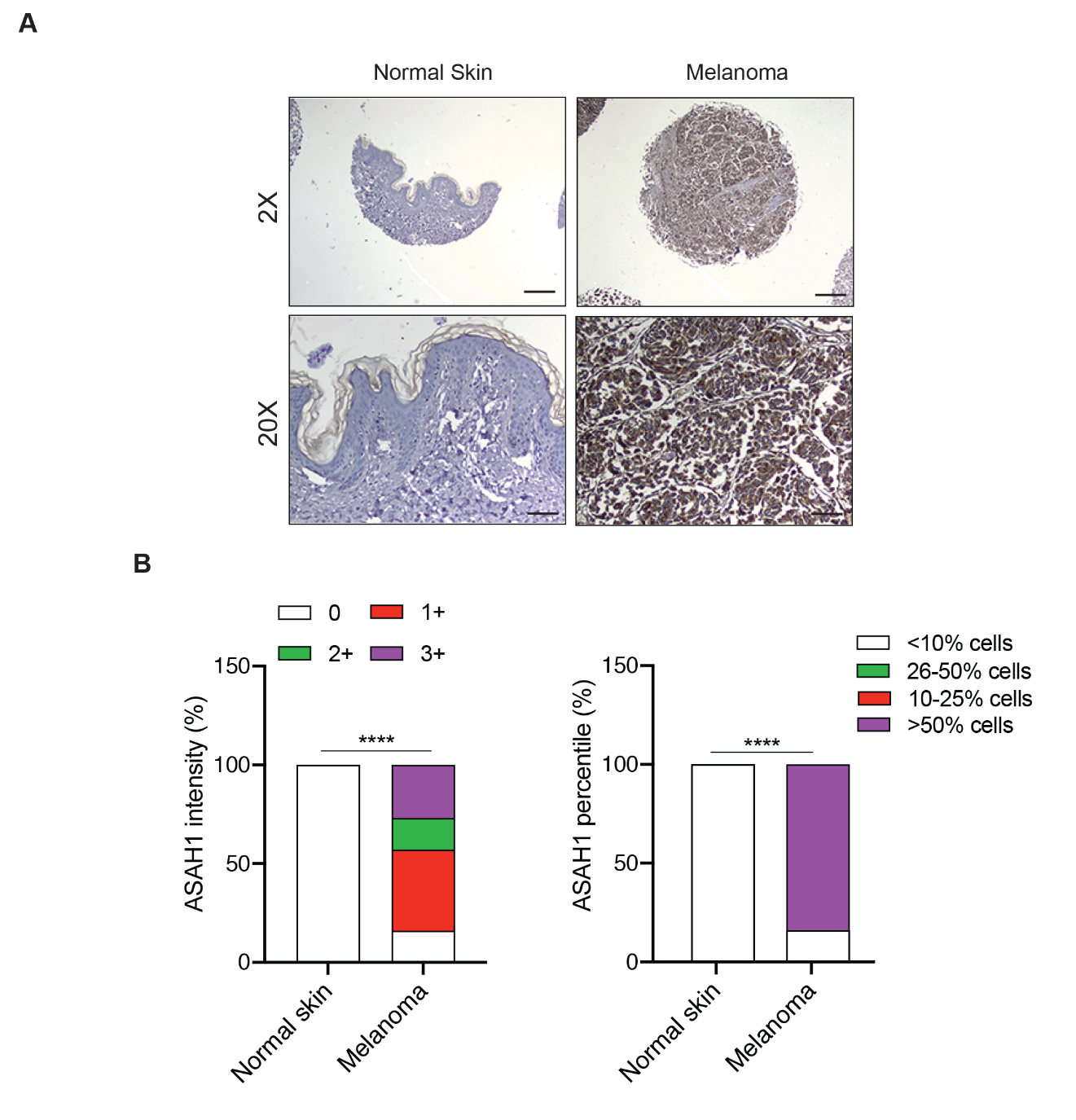
**

**Supplementary Figure 2. ASAH1 protein expression in melanoma patient samples.** (A) A tissue microarray (TMA, ME803b) of normal skin (n = 40) and melanoma samples (n = 37) was analyzed for ASAH1 protein expression. Representative images of ASAH1 immunohistochemical staining in normal skin or melanoma samples at 10× and 40× magnifications are shown. Scale bar: 500 μm for 2× and 50 μm for 20×. (B) Analysis of immunohistochemical data from the TMA with normal skin and melanoma samples. (Left) Normal skin and melanoma samples were scored 0, +1, +2, or +3 based on the ASAH1 staining intensity. A comparison of the average densities of ASAH1 staining in normal skin and melanoma samples is shown. (Right) Normal skin and melanoma samples were scored <10%, 10-25%, 26-50%, or >50% based on ASAH1 staining percentile. The comparison of average percentiles of ASAH1 expressing cells in normal skin and melanoma samples is shown. Contingency analysis using a chi-squared test was used to determine the significant difference in the ASAH1 expression between normal skin and melanoma samples. Data are presented as mean ± SEM; **** represents p values < 0.0001.

**
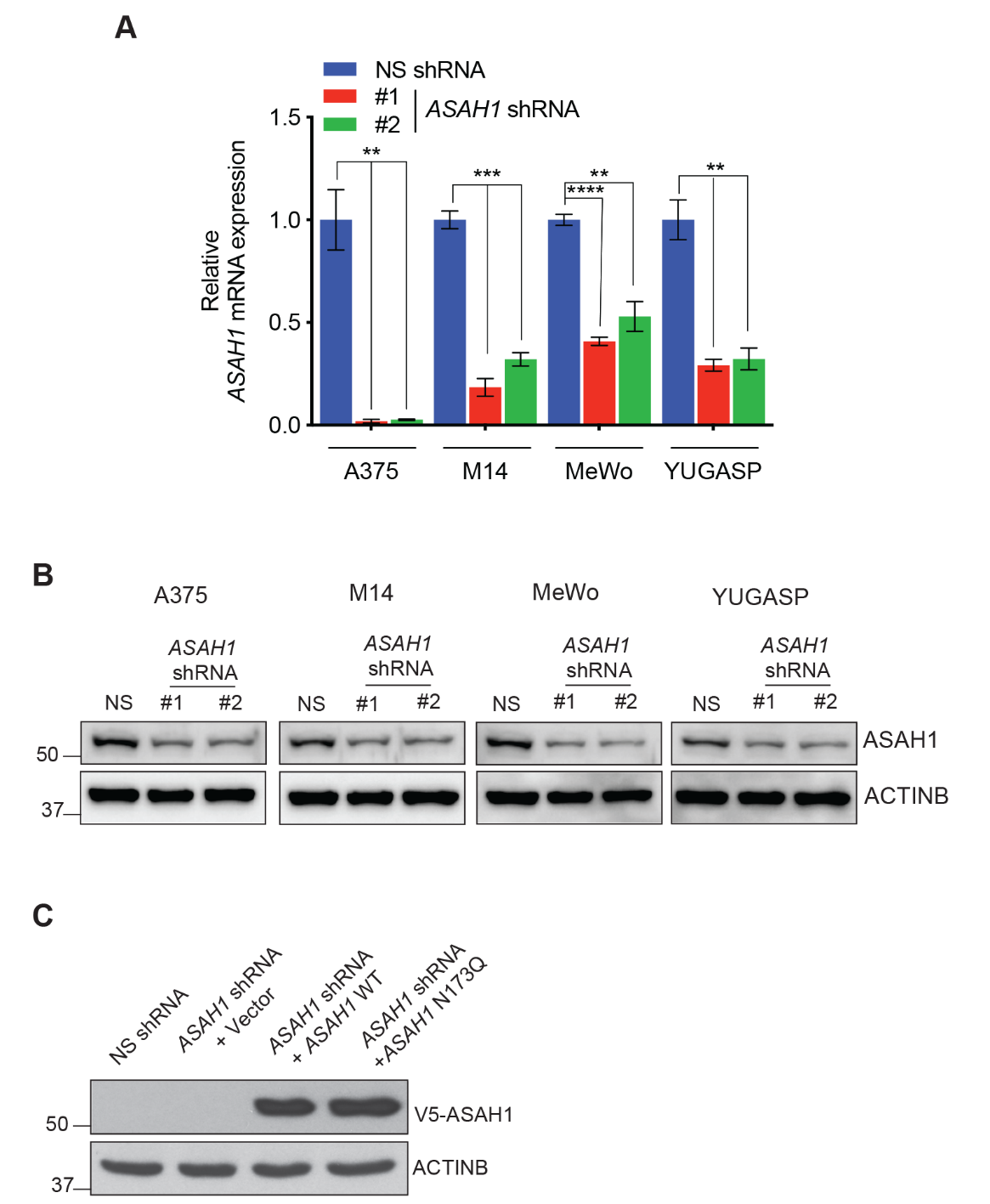
**

**Supplementary Figure 3. Knockdown and ectopic ASAH1 expression validation.** (A) The indicated melanoma cell lines expressing *ASAH1* shRNAs or NS shRNA were analyzed for the mRNA expression of *ASAH1* using RT-qPCR. Relative mRNA expression compared to NS shRNA-expressing cells is shown. (B) The indicated melanoma cell lines expressing *ASAH1* shRNAs or NS shRNA were analyzed for ASAH1 levels by immunoblotting. ACTINB was used as a loading control. (C) A375 cells expressing NS shRNA alone, *ASAH1* shRNA alone, or *ASAH1* shRNA with empty vector, wild-type *ASAH1*, or *ASAH1* N173Q were analyzed for the indicated proteins using immunoblotting. ACTINB was used as a loading control. Data are presented as mean ± SEM; **, ***, and **** represent p values < 0.01, < 0.001, and < 0.0001, respectively.

**
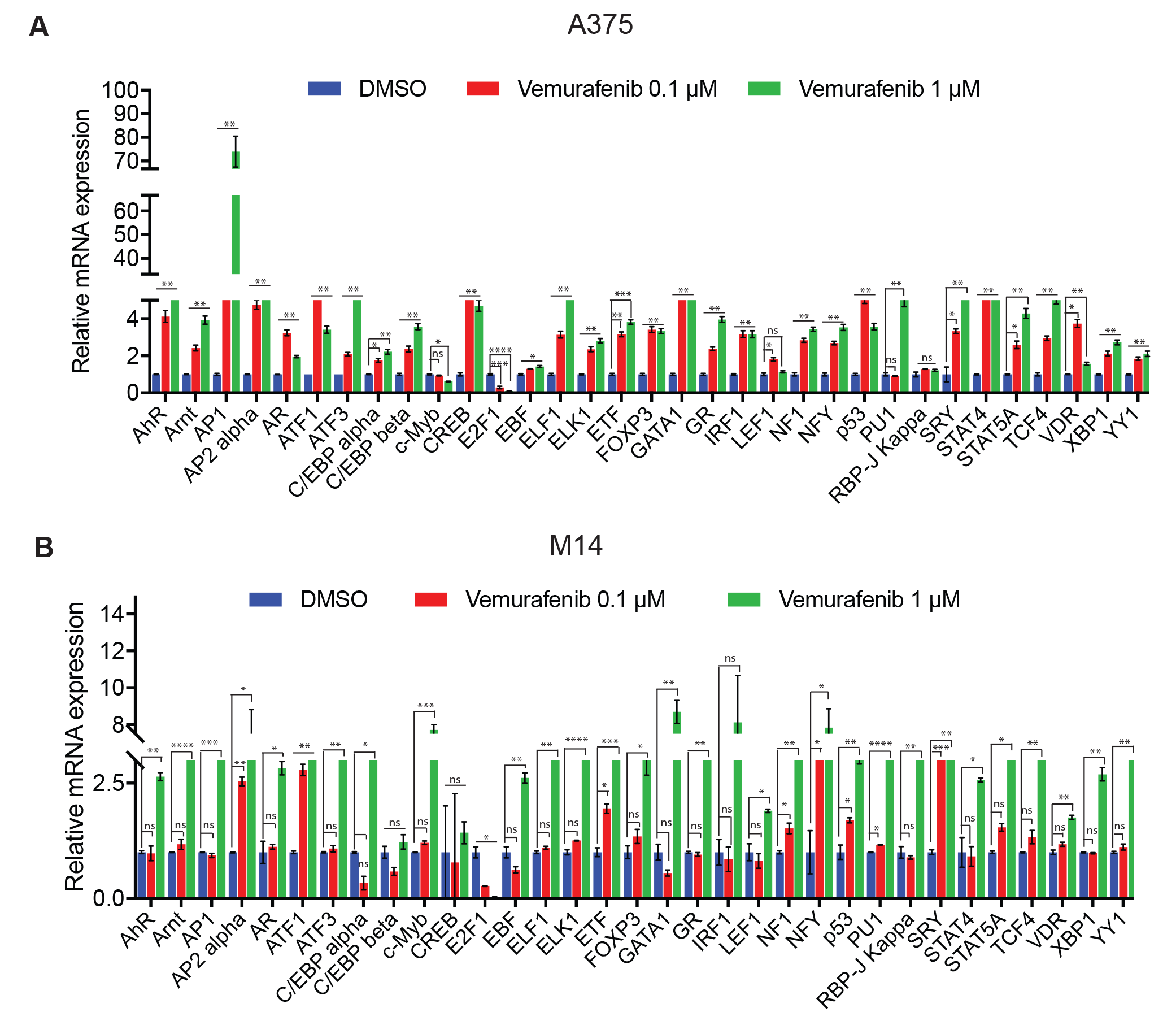
**

**Supplementary Figure 4. E2F1 was transcriptionally regulated by the MAPK pathway in melanoma cells.** (A-B) The indicated melanoma cell lines were treated with DMSO, 0.1 μM of vemurafenib, or 1.0 μM of vemurafenib for 24 h, and the mRNA expression of the indicated transcription factors was measured. mRNA expression of the indicated transcription factors was plotted relative to DMSO-treated cells. Data are presented as mean ± SEM; *, **, ***, and **** represent p values < 0.05, < 0.01, < 0.001, and < 0.0001, respectively.

**
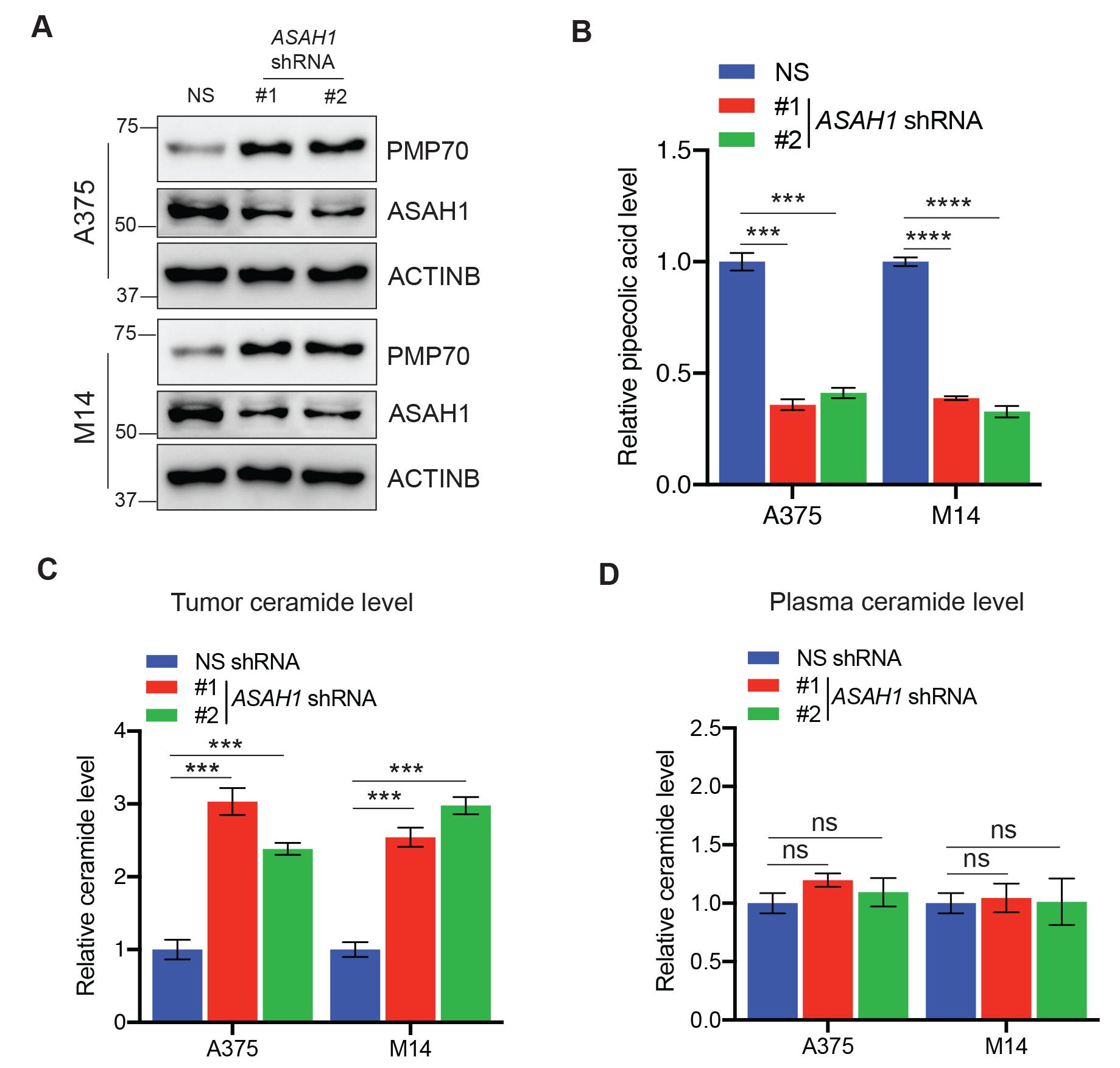
**

**Supplementary Figure 5.** **PMP70, pipecolic acid, and ceramide levels in ASAH1 knockdowns in vivo.** (A) The expression of the indicated proteins was measured in the tumor lysates from athymic nude mice that had been subcutaneously injected with indicated melanoma cell lines expressing either *ASAH1* shRNAs or NS shRNA using immunoblotting. ACTINB was used as a loading control. (B) The relative levels of pipecolic acid in the tumors from the athymic nude mice subcutaneously injected with the indicated melanoma cell lines expressing either *ASAH1* shRNAs or NS shRNA. (C) The relative ceramide levels in the tumors from the athymic nude mice subcutaneously injected with indicated melanoma cell lines expressing either *ASAH1* shRNAs or NS shRNA. (D) The relative ceramide levels in the plasma from the athymic nude mice subcutaneously injected with the indicated melanoma cell lines expressing either *ASAH1* shRNAs or NS shRNA. Data are presented as mean ± SEM; *** and **** represent p values < 0.001 and <0.0001, respectively; ns represents not significant p values.

**
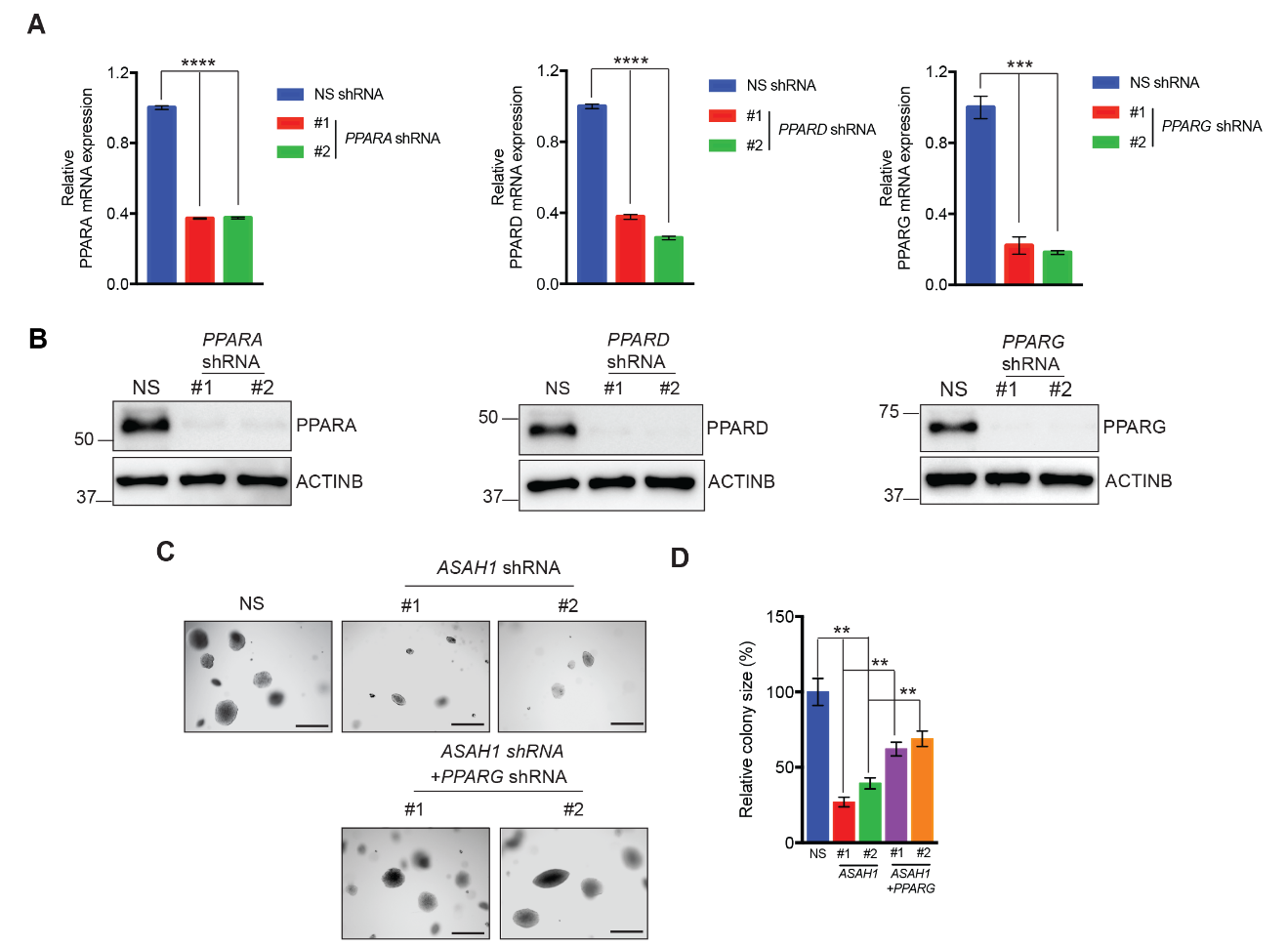
**

**Supplementary Figure 6. Knockdown of various PPAR isoforms and their role downstream of ASAH1.** (A) A375 cells expressing *PPARA*, *PPARD*, or *PPARG* shRNAs were analyzed for the mRNA expression of *PPARA*, *PPARD*, or *PPARG* using RT-qPCR. (B) A375 cells expressing *PPARA*, *PPARD*, or *PPARG* shRNAs were analyzed for protein expression of PPARα, PPARδ, and PPARγ by immunoblotting. ACTINB was used as a loading control. (C) A375 cells expressing NS shRNA alone, *ASAH1* shRNA alone, or *ASAH1* shRNA with *PPARG* shRNA were analyzed using a soft-agar assay. Representative images are shown. Scale bar, 500 μm. (D) Relative colony size from the experiment presented in panel C. Data are presented as mean ± SEM; *, **, ***, and **** represent p values < 0.05, < 0.01, < 0.001, and < 0.0001, respectively.

**
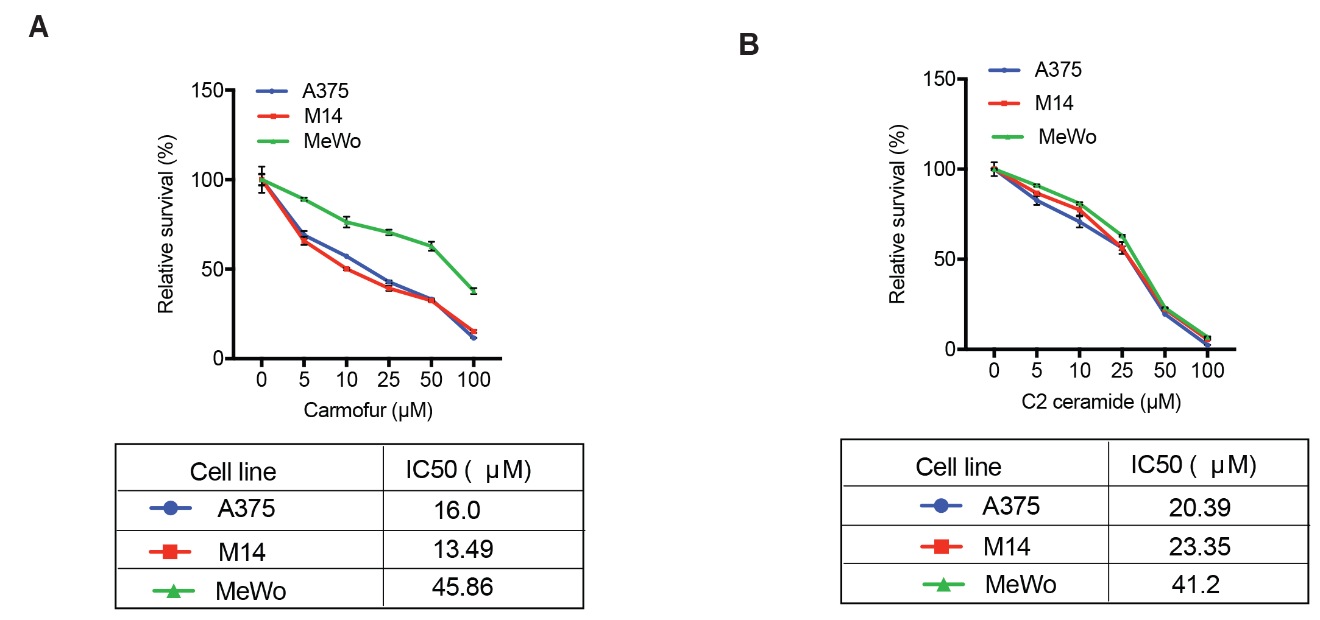
**

**Supplementary Figure 7. Treatment of melanoma cell lines with carmofur or C2 ceramide.** (A) (Top panel) The indicated melanoma cell lines were treated with the indicated concentrations of carmofur for 48 h. Survival was measured using an MTT assay. The percent relative survival compared with untreated cells is shown. (Bottom panel) IC_50_ values for the indicated cell lines are shown. (B) (Top panel) The indicated melanoma cell lines were treated with the indicated concentrations of C2 ceramide for 48 h. Cell survival was measured using an MTT assay. The percent relative survival compared with untreated cells is shown. (Bottom panel) IC_50_ values of the indicated cell lines are shown. Data are presented as mean ± SEM.

**
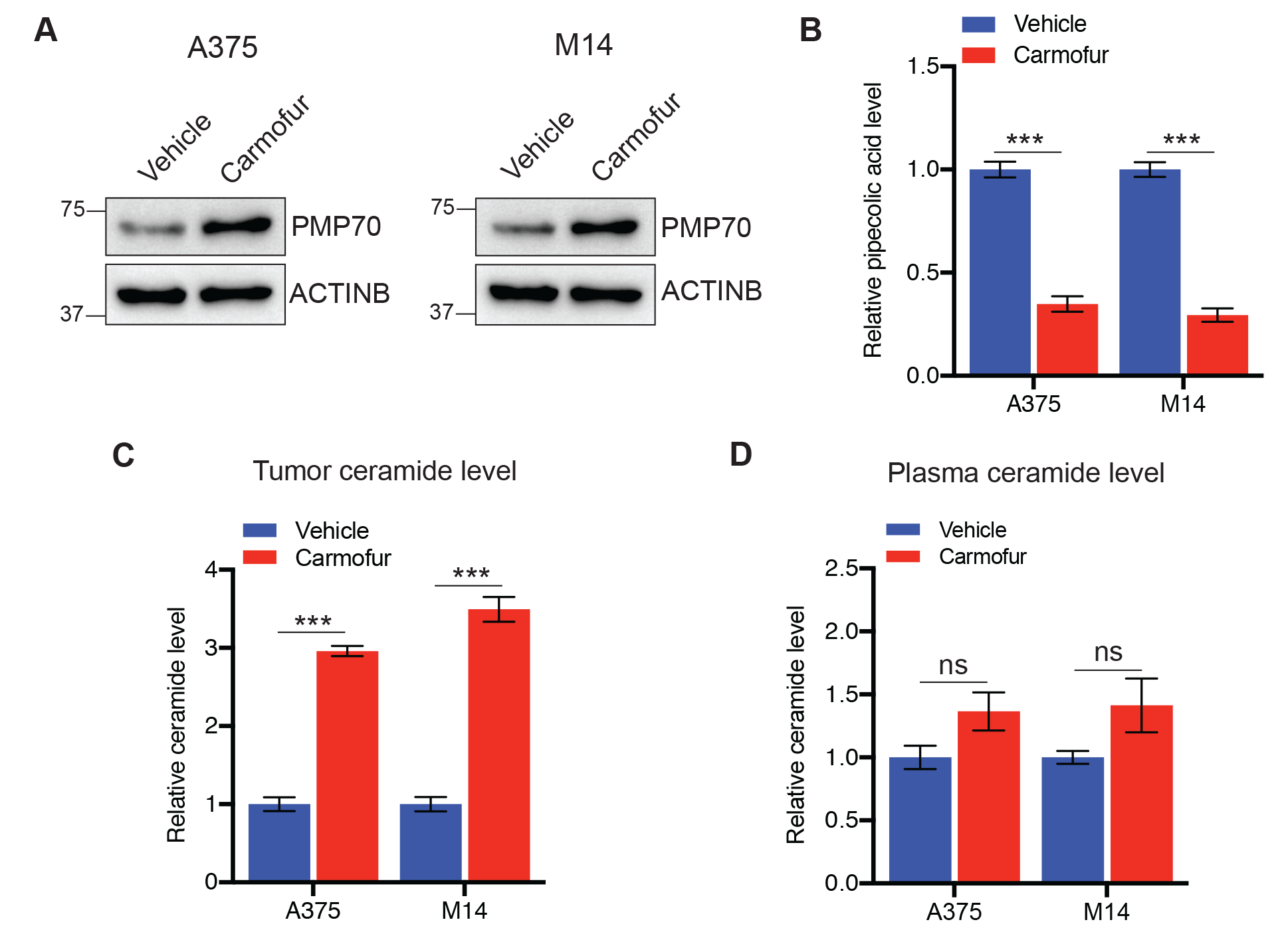
**

**Supplementary Figure 8. PMP70, pipecolic acid, and ceramide levels in vivo upon carmofur treatment**. (A) The expression of the indicated proteins was measured using immunoblotting in the tumor lysates from athymic nude mice subcutaneously injected with the indicated melanoma cell lines and treated orally with vehicle (0.5% methylcellulose) or carmofur (80 mg/kg). ACTINB was used as a loading control. (B) The relative levels of pipecolic acid in the tumors from the athymic nude mice subcutaneously injected with the indicated melanoma cell lines and treated orally with vehicle or carmofur. (C) The relative ceramide levels in the tumors from the athymic nude mice subcutaneously injected with the indicated melanoma cell lines and treated orally with vehicle or carmofur. (D) The relative ceramide levels in the plasma from the athymic nude mice subcutaneously injected with the indicated melanoma cell lines and treated orally with vehicle or carmofur. Data are presented as mean ± SEM; *** represents p values < 0.001; ns represents not significant p values.

**
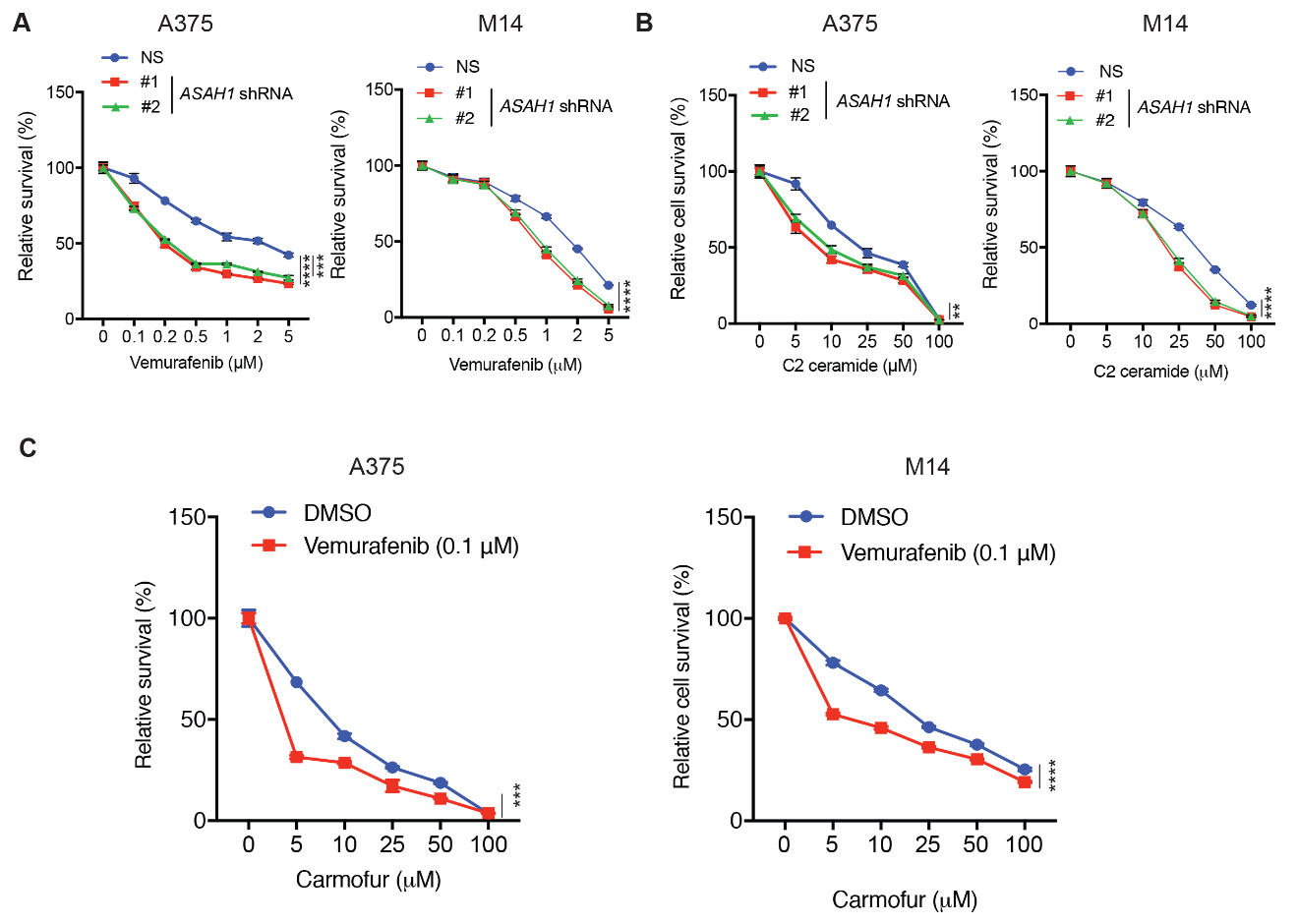
**

**Supplementary Figure 9. Pharmacological targeting of ASAH1 alone or in combination with vemurafenib**. (A) A375 or M14 cells expressing *ASAH1* shRNAs or NS shRNA were treated with the indicated concentrations of vemurafenib for 48 h. Survival was measured using an MTT assay. The percent relative survival compared with untreated cells is shown. (B) A375 or M14 cells expressing *ASAH1* shRNAs or NS shRNA were treated with the indicated concentrations of C2 ceramide for 48 h. Survival was measured using an MTT assay. The percent relative survival compared with untreated cells is shown. (C) A375 or M14 cells treated with DMSO or 0.1 μM of vemurafenib were also treated with the indicated concentrations of carmofur for 48 h. Cell survival was measured using an MTT assay. The percent relative survival compared with untreated cells is shown. Data are presented as mean ± SEM; *, **, ***, and **** represent p values < 0.05, < 0.01, < 0.001, and < 0.0001, respectively.

**SUPPLEMENTARY MATERIALS AND METHODS**

**Bioinformatic analysis of melanoma datasets**

The Haqq melanoma dataset [1], Talantov melanoma dataset [2], Riker melanoma dataset [3], Garnette cell-line dataset [4], and Wooster cell-line dataset were downloaded from Oncomine (https://www.oncomine.org), analyzed for *ASAH1* expression, and plotted as box plots comparing melanoma with normal skin samples and different melanoma stages and different melanoma genotypes. The Haqq melanoma dataset analyzed 25 melanomas, 9 non-neoplastic nevi, and 3 normal skin samples using a cDNA microarray. The Talantov melanoma dataset analyzed 45 cutaneous melanomas, 18 benign melanocytic skin nevi, and 7 normal skin samples using an Affymetrix U133A microarray. The Riker melanoma dataset analyzed 40 metastatic melanomas, 42 primary melanomas, 4 normal skin samples, and 1 normal skin primary culture using an Affymetrix HG U133 Plus 2.0 microarray. The Garnett cell-line dataset analyzed 732 cell lines. Data include microsatellite status, information on 64 mutated genes and 7 gene fusions, and drug sensitivities to numerous targeted and chemotherapy drugs. This dataset was created by performing robust multi-array averaging on CEL files from the Broad Institute; replicates were averaged when present. The Wooster cell-line dataset analyzed 318 cell lines submitted to caArray by GlaxoSmithKline using an Affymetrix U133 Plus 2.0 microarray. Sample data included cell-line name, disease, tissue, and drug sensitivity. In addition, gIC_50_ values for 19 drugs in 228 cell lines were obtained from Greshock et al. [5]. Compendia Bioscience determined the drug sensitivity of the cell lines by applying a standard visual inspection protocol to the gIC_50_ distributions. The corresponding DNA copy number data are available in Wooster CellLine 2. To analyze the expression of *ASAH1* in the Cancer Genome Atlas (TCGA) dataset, we used Gene Expression Profiling Interactive Analysis (GEPIA) at http://gepia.cancer-pku.cn. *ASAH1* was analyzed in 461 melanoma samples and 558 normal skin samples (Match TCGA + GTEx data).

#### Chromatin immunoprecipitation

The *ASAH1* promoter sequence was downloaded from the UCSC genome browser and analyzed using PROMO 3.0 and rVista 2.0 software. Chromatin immunoprecipitation experiments were performed as previously described (Gazin et al., 2007). Normalized Ct (ΔCt) values were calculated by subtracting the Ct of input DNA from that of immunoprecipitated DNA (ΔCt = Ct[IP] - Ct[input]). The relative fold enrichment of a factor at the target site was calculated using the formula 2^-(ΔCt(T)-ΔCt(Actb))^, where ΔCt(T) and ΔCt(Actb) are ΔCt values of the target and β-actin (negative control) primers, respectively.

**Soft-agar assay**

Soft-agar assays were performed by seeding 5 × 10^3^ melanoma cells stably expressing the indicated shRNA or cDNA constructs onto 0.4% low-melting-point agarose (Sigma-Aldrich) layered on top of 0.8% agarose. After 3-4 weeks of incubation, colonies were stained with a 0.005% crystal violet solution and imaged using a microscope. Colony size was measured using microscopy and plotted as percent relative colony size compared with control cells. Colony sizes were quantified using ImageJ software (https://imagej.nih.gov/ij/). Statistical analysis was performed using Student’s *t* test in GraphPad Prism 7 software.

**MTT assay**

Melanoma cells were plated at a density of 5 × 10^3^ cells/well in 96-well plates and allowed to adhere for 24 h. The cells were then treated with vehicle, inhibitors, or drugs depending on the experimental conditions. Following treatment, the medium was removed, and 20 μl of methylthiazole tetrazolium (MTT; 5 mg/ml in phosphate-buffered saline [PBS]; Sigma-Aldrich, St. Louis, MO, USA) was added and incubated for 2 h at 37°C. Formazan crystals were solubilized in 100 μl of DMSO and the absorbance was measured at 570 nm with a reference wavelength of 630 nm.

**Plasmids and cloning**

LentiORF-ASAH1 expression vector was obtained from GE Dharmacon (Clone ID: ccsbBroad304_05857). Amino acid N173 in ASAH1 is a glycosylation site. We performed site-directed mutagenesis to generate ASAH1 N173Q, which abolishes ASAH1 activity, using a QuikChange II XL Site-Directed Mutagenesis kit (Agilent) per the manufacturer’s protocol. The PPRE X3-TK-luc vector was a gift from Bruce Spiegelman (Plasmid #1015, Addgene) and was previously described (Kim et al., 1998).

#### RNA preparation, cDNA synthesis, and RT-qPCR analysis

Total RNA was extracted using TRIzol (Invitrogen) and purified using an RNeasy Mini kit (Qiagen) according to the manufacturer’s instructions. cDNA was generated using a ProtoScript first-strand cDNA synthesis kit (New England Biolabs). Quantitative PCR was performed using Power SYBR Green Master Mix (Life Technologies). The oligonucleotide sequences used for RT-qPCR are provided in **Supplementary Table 1**.

**Immunoblotting**

Cells were washed with ice-cold PBS and lysed using ice-cold IP lysis buffer (Thermo Fisher Scientific, Waltham, MA, USA) containing protease inhibitor (Roche, USA) and phosphatase inhibitor cocktail (Sigma-Aldrich, St. Louis, MO, USA). Briefly, lysed samples were centrifuged at 12000 rpm for 40 min, and clarified supernatants were stored at -80 °C. Protein concentrations were determined using Bradford Protein Assay Reagent (Bio-Rad, Hercules, CA, USA). Equal amounts of protein samples (50-100 μg) were electrophoresed on 6-12% SDS-polyacrylamide gels and transferred onto polyvinylidene difluoride membranes (Millipore, Burlington, MA, USA). The membranes were blocked and probed with primary antibodies. After washing, the membranes were incubated with appropriate horseradish peroxidase (HRP)-conjugated secondary antibodies (1:2000) (GE Healthcare Bio-Sciences, Marlborough, MA, USA), and blots were developed using luminescence detection reagents (Thermo Fisher Scientific). The antibodies used for immunoblotting are listed in **Supplementary Table 1**.

**Matrigel invasion assay**

The invasion assay was performed in a BioCoat Growth Factor Reduced Matrigel Invasion Chamber (Cat# 354483, BD Biosciences) using A375 or M14 cells expressing *ASAH1* or non-specific shRNAs. Briefly, the cells were serum starved for 6 h and then 5 × 10^4^ cells/insert were seeded in triplicate in low-serum medium. The cells were incubated for 20 h to allow them to invade toward serum-rich medium in the bottom well. The number of cells invading the Matrigel was quantified by DAPI staining and imaging; 8-12 fields per membrane were counted, and nuclei quantification was performed using ImageJ.

**Wound-healing assay**

For the wound-healing assay, A375 cells expressing *ASAH1* or non-specific shRNA were grown in 12-well plates until fully confluent. A scratch was created using a sterile 20-µl pipette tip, and cell migration into the wound was monitored at 0, 12, and 24 h using light microscopy. The quantification of wound healing was performed using ImageJ.

**Clonogenic assay**

Approximately 2 × 10^6^ cells were plated in 100-mm cell culture dishes and allowed to grow for 48 h. The cells were treated with 2 μM of vemurafenib alone or in combination with 5 μM of carmofur over four weeks. The medium was changed every 3 days with drug treatment. After 4 weeks, the cells were stained with a 0.005% Coomassie Brilliant Blue R-250 solution (Bio-Rad, USA), and the plates were imaged using an Epson Perfection V800 Photo Scanner.

**SUPPLEMENTARY REFERENCES**

[1] Haqq, C., Nosrati, M., Sudilovsky, D., Crothers, J., Khodabakhsh, D., Pulliam, B.L., et al., 2005. The gene expression signatures of melanoma progression. Proc Natl Acad Sci U S A 102(17):6092-6097.

[2] Talantov, D., Mazumder, A., Yu, J.X., Briggs, T., Jiang, Y., Backus, J., et al., 2005. Novel genes associated with malignant melanoma but not benign melanocytic lesions. Clin Cancer Res 11(20):7234-7242.

[3] Riker, A.I., Enkemann, S.A., Fodstad, O., Liu, S., Ren, S., Morris, C., et al., 2008. The gene expression profiles of primary and metastatic melanoma yields a transition point of tumor progression and metastasis. BMC Med Genomics 1:13.

[4] Garnett, M.J., Edelman, E.J., Heidorn, S.J., Greenman, C.D., Dastur, A., Lau, K.W., et al., 2012. Systematic identification of genomic markers of drug sensitivity in cancer cells. Nature 483(7391):570-575.

[5] Greshock, J., Bachman, K.E., Degenhardt, Y.Y., Jing, J., Wen, Y.H., Eastman, S., et al., 2010. Molecular target class is predictive of in vitro response profile. Cancer Res 70(9):3677-3686.
