# Supplementary material for: N-acylsphingosine amidohydrolase 1 promotes melanoma growth and metastasis by suppressing peroxisome biogenesis-induced ROS production
Source: Mol Metab. 2021 Mar 23;48:101217. doi: 10.1016/j.molmet.2021.101217 (PMC8081993; doi:10.1016/j.molmet.2021.101217)
Supplement: Supplementary file 2 — Multimedia component 2 [file mmc2.docx]

**Supplementary Table 1.** Key resource table: Primer sequences for RT-qPCR analysis, clone ID and catalog numbers for shRNAs (Open Biosystems), antibodies, source, and concentration of chemical inhibitors

| **Application** | **Gene symbol** | **Forward primer (5′-3′)** | **Reverse primer (5′-3′)** |
| --- | --- | --- | --- |
| **RT-qPCR** | ASAH1 | AGTTGCGTCGCCTTAGTCCT | TGCACCTCTGTACGTTGGTC |
|  | PPARG | ggagcctgcatctccacctt | ccggaagaaacccttgcatc |
|  | PPARA | ccaggagaacatgcccacag | acacccagcaggccctacat |
|  | PPARD | tggctttgtcacccgtgagt | gccggtctccacacagaatg |
|  | ABCD3 | ttcagctccctgacctgctg | atgtgcatgggtccacaagg |
|  | AhR | gcagcgccaacatcacctac | ggctagccaaacggtccaac |
|  | Arnt | aggataaggggtgggggaga | agcaggggaacagccagaag |
|  | AP-1 | ttcaggaggctggaggaagg | tggtcacagcacatgccact |
|  | AP-2 alphaA | ttacctcacgccatcgagga | ggcattgctgttggacttgg |
|  | AR | ggcggggtaagggaagtagg | cccgggttctggatcacttc |
|  | ATF1 | tagcctaccccaaacgcaca | taccgtttcctcggcagtca |
|  | ATF3 | tctgcctcggaagtgagtgc | ttccttgacaaagggcgtca |
|  | C/EBP alpha | tcccagagggaccggagtta | cattgcacaaggcactgctg |
|  | C/EBP beta | ggacaagcacagcgacgagt | cttgtgctgcgtctccaggt |
|  | c-Myb | acggtccgaaacgttggtct | tgcgatttctgcccatctgt |
|  | CREB | cacgcctgtaatcccagcac | ctgctgcctcaacctcctga |
|  | E2F1 | ccccctggatttctgacagg | gcctcctagcggtagccaga |
|  | EBF | ctcaaaagggctgcggatct | ggccagttgtccactgaacg |
|  | ELF-1 | agattccccagccactacgc | tcgctgggtccacttgatgt |
|  | Elk-1 | ggccatactcaggggtccag | cctccctggccaaaaagttg |
|  | ETF | actgatcgcccgctacatca | ctgggcagaggacatggttg |
|  | FOXP3 | ttcacctacgccacgctcat | gactcaggttgtggcggatg |
|  | GATA-1 | ggggttttcttcccctctgg | ggcctcagcgtccctgtagt |
|  | GR | acaggcagcgatggtctcag | ctgggtcagagcctcagcaa |
|  | IRF-1 | tgagccgagaacatgccatt | ggccaggctggtctcaaact |
|  | LEF-1 | ccaggctggtctgcaagaga | tgcacgttgggaatgagctt |
|  | NF-1 | ccatggtcctctcccaaagg | gagaaggttgccccatgtcc |
|  | NF-Y | ccttacggtcgctgggaatc | gccagggcaggtctactgct |
|  | p53 | accggagtcattgggaagga | gccttctctgtcccccagaa |
|  | PU-1 | cagcccagctcagatgagga | ttgtccacccaccagatgct |
|  | RBP-J kappa | tgcattccgagaaggttgga | gattcgttagggggcacctg |
|  | SRY | atcccgcttcggtactctgc | ggcctagctggtgctccatt |
|  | STAT4 | ttcccacaaaagcagctcca | ttccctgccaccttgctgta |
|  | STAT5A | caagagtgcgccgagtctgt | acctgtctcccctccccttc |
|  | TCF-4 | atggaaccggccttctttca | tcgcagactggacaggaagc |
|  | VDR | cccaagctgtctgaggagca | ggctccctccaccatcattc |
|  | XBP-1 | cccatggattctggcggtat | ctggggaagggcatttgaag |
|  | YY1 | cccagggcaggaatgaaaag | aagcgtttcccacagccttc |
|  | ACTINB | gcatggagtcctgtggcatc | ttctgcatcctgtcggcaat |
|  | | | |
| **ChIP** |  | **Forward primer** | **Reverse primer** |
|  | ASAH1  promoter E2F1  binding | CTGCTCCTTGTCCCTGACTC | CACGAAACGGATCCAAAGAG |
|  | ACTINB | GAGGGGAGAGGGGGTAAAAA | AAAGGCGAGGCTCTGTGCT |
|  | GAPDH | TCCGGGTGATGCTTTTCCTAG | TTTGCGGTGGAAATGTCCTTTTC |
|  | | | |
| **Sequencing** | **Name** | **Forward primer** | **Reverse primer** |
|  | pLX304-Blast-V5 | CGCAAATGGGCGGTAGGCGTG | TACGGGAAGCAATAGCATGA |
|  |  |  |  |
| **Site-directed mutagenesis** | **Mutation** | **Forward primer (5′-3′)** | **Reverse primer (5′-3′)** |
|  | ASAH1 N173Q | TTGGGTGGAACATAAAT**CAA**GATACCTGGGTCATAAC | GTTATGACCCAGGTATC**TTG**ATTTATGTTCCACCCAA |
|  |  |  |  |
|  | | | |
| **shRNAs** | **Gene symbol** | **Clone ID** | **Catalog number** |
|  | *ASAH1* | RHS3979-9596807 | TRCN0000029399 |
|  | *ASAH1* | RHS3979-9596811 | TRCN0000029403 |
|  | *E2F1* | RHS3979-9568610 | TRCN0000000250 |
|  | *E2F1* | RHS3979-9568611 | TRCN0000000251 |
|  | *PPARG* | RHS3979-9570179 | TRCN0000001672 |
|  | *PPARG* | RHS3979-9570181 | TRCN0000001674 |
|  | *PPARA* | RHS3979-9570172 | TRCN0000001665 |
|  | *PPARA* | RHS3979-9570173 | TRCN0000001666 |
|  | *PPARD* | RHS3979-9570168 | TRCN0000001662 |
|  | *PPARD* | RHS3979-9570170 | TRCN0000001663 |
|  | | | |
| **Immunoblotting** | **Protein symbol** | **Antibody source** | **Dilution** |
|  | ASAH1 | Santa Cruz Biotechnology  (Cat# sc-292176) | 1:1000 |
|  | Phospho-ERK | Cell Signaling (Cat# 4376) | 1:1000 |
|  | Total-ERK | Cell Signaling (Cat# 4695) | 1:1000 |
|  | E2F1 | Santa Cruz Biotechnology  (Cat# sc-251) | 1:500 |
|  | PPARA | Santa Cruz Biotechnology  (Cat# sc-398394) | 1:500 |
|  | PPARD | Santa Cruz Biotechnology  (Cat# sc-74517) | 1:500 |
|  | PPARG | Santa Cruz Biotechnology  (Cat# sc-271392) | 1:500 |
|  | V5 | Cell Signaling (Cat# 13202) | 1:1000 |
|  | ACTINB | Cell Signaling (Cat# 4970) | 1:2500 |
|  |  |  |  |
| **Immunofluorescence** | **Protein symbol** | **Antibody source** | **Dilution** |
|  | PMP70 | Invitrogen (Cat# PA1-650) | 1:1000 |
|  | | | |
| **IHC** | **Protein symbol** | **Antibody source** | **Dilution** |
|  | ASAH1 | Sigma-Aldrich (Cat# HPA005468) | 1:100 |
|  |  |  |  |
|  | | | |
| **Pharmacological treatment** | **Compounds** | **Concentration** | **Source** |
|  | Vemurafenib | Indicated concentrations | Selleck Chemicals |
|  | Rosiglitazone | Indicated concentrations | Sigma-Aldrich |
|  | C2 ceramide | Indicated concentrations | Sigma-Aldrich |
|  | Carmofur | Indicated concentrations | Sigma-Aldrich or Cayman Chemical |
