# Supplementary material for: N-acylsphingosine amidohydrolase 1 promotes melanoma growth and metastasis by suppressing peroxisome biogenesis-induced ROS production
Source: Mol Metab. 2021 Mar 23;48:101217. doi: 10.1016/j.molmet.2021.101217 (PMC8081993; doi:10.1016/j.molmet.2021.101217)
Supplement: Supplementary file 3 — Multimedia component 3 [file mmc3.docx]

**Supplementary Table 2.** Summary of immunohistochemistry staining for ASAH1 in human normal skin and melanoma samples

| **TMA** | | **ME2081** | | | | | | | | | | | |
| --- | --- | --- | --- | --- | --- | --- | --- | --- | --- | --- | --- | --- | --- |
| **Staining** | | **Cytoplasmic** | | | | | | | | | | | |
| Density | | 0 | | | 1+ | | 2+ | | | | 3+ | | |
| Percentile | | < 10% | | | 11-25% | | 26-50% | | | | < 50% | | |
|  | | | | | | | | | | | | | |
| **Tissue** | **Normal skin** | | | | | | | | | | | | |
| **Tissue ID** | **Age** | **Sex** | **Organ (anatomic site)** | **Pathology diagnosis** | | **TNM** | | **Grade** | **Stage** | **Type** | | **Density** | **Percentile** |
| Kin08N033A005 | 42 | F | Skin | Normal dermatic tissue (chronic inflammation) of scalp | | - | | - | - | Normal | | 0 | 0 |
| 06N26 | 40 | F | Skin | Normal dermatic tissue of abdominal part | | - | | - | - | Normal | | 0 | 0 |
| 07N26 | 30 | M | Skin | Normal dermatic tissue of abdominal part | | - | | - | - | Normal | | 0 | 0 |
| 07N034 | 40 | F | Skin | Normal dermatic tissue of abdominal part | | - | | - | - | Normal | | 0 | 0 |
| 06N27 | 19 | M | Skin | Normal dermatic tissue of abdominal part | | - | | - | - | Normal | | 0 | 0 |
| Kin08N034A007 | 50 | F | Skin | Normal dermatic tissue of abdominal part | | - | | - | - | Normal | | 0 | 0 |
| Kin07N004A001 | 28 | M | Skin | Normal dermatic tissue of abdominal part | | - | | - | - | Normal | | 0 | 0 |
| Kin06N27A001 | 19 | M | Skin | Normal dermatic tissue of abdominal part | | - | | - | - | Normal | | 0 | 0 |
| Kin06N019A001 | 43 | M | Skin | Normal dermatic tissue of thigh | | - | | - | - | Normal | | 0 | 0 |
| Kin07N015A002 | 35 | M | Skin | Normal dermatic tissue | | - | | - | - | Normal | | 0 | 0 |
| Kin06N023A002 | 21 | F | Skin | Normal dermatic tissue of abdominal part | | - | | - | - | Normal | | 0 | 0 |
| Kin06N025A001 | 1 | M | Skin | Normal dermatic tissue of scalp | | - | | - | - | Normal | | 0 | 0 |
| Kin07N001A001 | 41 | F | Skin | Normal dermatic tissue of scalp | | - | | - | - | Normal | | 0 | 0 |
| Kin06N024A001 | 21 | F | Skin | Normal dermatic tissue of scalp | | - | | - | - | Normal | | 0 | 0 |
| Kin08N040A001 | 16 | F | Skin | Normal dermatic tissue of abdominal part | | - | | - | - | Normal | | 0 | 0 |
| Kin07N0020A001 | 40 | F | Skin | Normal dermatic tissue of abdominal part | | - | | - | - | Normal | | 0 | 0 |
|  | | | | | | | | | | | | | |
| **Tissue** | **Melanoma** | | | | | | | | | | | | |
| **Tissue ID** | **Age** | **Sex** | **Organ (anatomic site)** | **Pathology diagnosis** | | **TNM** | | **Grade** | **Stage** | **Type** | | **Density** | **Percentile** |
| Fvl041282B001 | 41 | F | Vulva | Malignant melanoma (sparse) | | T4N0M0 | | - | II | Malignant | | 0 | 0 |
| Fvl050429B001 | 38 | F | Vulva | Malignant melanoma | | T4N0M0 | | - | II | Malignant | | 1+ | 30% |
| Fvl030086B001 | 57 | F | Vulva | Malignant melanoma | | T4N0M0 | | - | II | Malignant | | 0 | 0 |
| Fvl060506B001 | 49 | F | Vulva | Malignant melanoma | | T4N1M0 | | - | III | Malignant | | 0 | 0 |
| Fvl041203B001 | 62 | F | Vagina | Malignant melanoma | | T4N0M0 | | - | II | Malignant | | 2+ | 60% |
| Fvl041118B001 | 34 | F | Vulva | Malignant melanoma | | T4N0M0 | | - | II | Malignant | | 1+ | 30% |
| Fvl040938B001 | 15 | F | Clitoris | Malignant melanoma | | T4N0M0 | | - | II | Malignant | | 0 | 0 |
| Dre030434B002 | 70 | F | Anus | Malignant melanoma | | - | | - | - | Malignant | | 3+ | 90% |
| Dre030138B001 | 67 | F | Rectum | Malignant melanoma | | - | | - | - | Malignant | | 0 | 0 |
| Dre051429B001 | 69 | F | Rectum | Malignant melanoma | | - | | - | - | Malignant | | 0 | 0 |
| Dre041810B001 | 72 | F | Rectum | Malignant melanoma | | - | | - | - | Malignant | | 1+ | 30% |
| Dre061446B001 | 57 | M | Rectum | Malignant melanoma | | - | | - | - | Malignant | | 2+ | 90% |
| Dre062768B001 | 67 | M | Rectum | Malignant melanoma (sparse) with necrosis | | - | | - | - | Malignant | | 2+ | 70% |
| Dre061890B001 | 42 | M | Anal canal | Malignant melanoma | | - | | - | - | Malignant | | 3+ | 90% |
| Dre031701B001 | 84 | F | Crissum | Malignant melanoma | | - | | - | - | Malignant | | 0 | 0 |
| Dre023410B001 | 64 | F | Rectum | Malignant melanoma | | - | | - | - | Malignant | | 0 | 0 |
| Dre010332B001 | 38 | F | Rectum | Malignant melanoma (sparse) with necrosis | | - | | - | - | Malignant | | 0 | 0 |
| Dre061865B001 | 52 | F | Anal canal | Malignant melanoma | | - | | - | - | Malignant | | 0 | 0 |
| Dre062316B001 | 47 | F | Rectum | Malignant melanoma | | - | | - | - | Malignant | | 1+ | 5% |
| Dre031567B001 | 66 | M | Rectum | Malignant melanoma | | - | | - | - | Malignant | | 1+ | 5% |
| Dre041097B001 | 54 | F | Anal canal | Malignant melanoma | | - | | - | - | Malignant | | 1+ | 20% |
| Dre041512B001 | 52 | F | Rectum | Malignant melanoma | | - | | - | - | Malignant | | 0 | 0 |
| Dre062643B001 | 75 | M | Rectum | Malignant melanoma (smooth muscle) | | - | | - | - | Malignant | | 0 | 0 |
| Kin060183B001 | 59 | F | Anus | Malignant melanoma | | - | | - | - | Malignant | | 1+ | 20% |
| Des050286B001 | 64 | M | Esophagus | Malignant melanoma | | - | | - | - | Malignant | | 0 | 0 |
| Dst010327B001 | 55 | M | Stomach | Malignant melanoma | | - | | - | - | Malignant | | 1+ | 5% |
| Din024558B001 | 71 | M | Small intestine | Malignant melanoma | | - | | - | - | Malignant | | 1+ | 5% |
| Doc051685B001 | 40 | M | Gingiva | Malignant melanoma of right submaxilla | | - | | - | - | Malignant | | 0 | 0 |
| Doc024058B001 | 36 | F | Oral cavity | Malignant melanoma | | - | | - | - | Malignant | | 0 | 0 |
| 181423B5 | 60 | F | Right jaw bones | Malignant melanoma | | T4N0M0 | | - | II | Malignant | | 3+ | 90% |
| 181422B1 | 60 | F | Right jaw bones | Malignant melanoma | | T4N0M0 | | - | II | Malignant | | 3+ | 90% |
| Atc060179B001 | 40 | M | Skin | Malignant melanoma of right chest wall | | T4N0M0 | | - | II | Malignant | | 0 | 0 |
| Kin040084B001 | 65 | M | Skin | Malignant melanoma of right sole of foot | | TxN0M0 | | - | - | Malignant | | 0 | 0 |
| Kin040100B001 | 31 | M | Skin | Malignant melanoma of scalp | | T4N0M0 | | - | II | Malignant | | 0 | 0 |
| Kin060124B001 | 80 | M | Skin | Malignant melanoma of right sole of foot | | T4N0M0 | | - | II | Malignant | | 1+ | 10% |
| Kin060148B001 | 51 | M | Skin | Malignant melanoma of chest wall | | T4N0M0 | | - | II | Malignant | | 0 | 0 |
| Kin060062B001 | 55 | M | Skin | Malignant melanoma of right upper arm | | T4N0M0 | | - | II | Malignant | | 0 | 0 |
| Kin060160B001 | 52 | F | Skin | Malignant melanoma of left heel | | T3N0M0 | | - | II | Malignant | | 0 | 0 |
| Kin060042B001 | 56 | F | Skin | Malignant melanoma of left leg | | T3N0M0 | | - | II | Malignant | | 3+ | 80% |
| Kin060059B001 | 36 | M | Skin | Malignant melanoma of right chest wall | | T4aN0M0 | | - | II | Malignant | | 0 | 0 |
| Kin060087B001 | 53 | F | Skin | Malignant melanoma of back | | T3N0M0 | | - | II | Malignant | | 0 | 0 |
| Kin060158B001 | 52 | M | Skin | Malignant melanoma of abdominal part | | T4N0M0 | | - | II | Malignant | | 3+ | 80% |
| Kin060085B001 | 78 | M | Skin | Malignant melanoma of right leg | | T4bN0M0 | | - | II | Malignant | | 1+ | 30% |
| Kin060043B001 | 42 | M | Skin | Malignant melanoma of right sole of foot | | T2N0M0 | | - | I | Malignant | | 2+ | 80% |
| Kin060149B001 | 51 | M | Skin | Malignant melanoma of left upper arm | | T4N1M0 | | - | III | Malignant | | 0 | 0 |
| Kin060094B001 | 70 | M | Skin | Malignant melanoma (sparse) of left face | | T3N0M0 | | - | II | Malignant | | 0 | 0 |
| Kin060058B001 | 51 | M | Skin | Malignant melanoma of left oxter | | T4N0M0 | | - | II | Malignant | | 0 | 0 |
| Kin050114B001 | 51 | M | Skin | Malignant melanoma of back | | T4N0M0 | | - | II | Malignant | | 0 | 0 |
| Kin060180B001 | 61 | M | Skin | Malignant melanoma of right groin | | T4N2M0 | | - | III | Malignant | | 0 | 0 |
| Kin060063B002 | 61 | M | Skin | Malignant melanoma (sparse) with hemorrhage of left oxter | | T4bN0M1 | | - | IV | Malignant | | 2+ | 60% |
| Kin040136B001 | 16 | F | Skin | Malignant melanoma of back | | T4N0M0 | | - | II | Malignant | | 2+ | 60% |
| Kin040177B001 | 27 | M | Skin | Malignant melanoma of right thigh | | T3N1M0 | | - | III | Malignant | | 2+ | 60% |
| Kin050001B001 | 63 | F | Skin | Malignant melanoma of right heel | | T4N0M0 | | - | II | Malignant | | 0 | 0 |
| Kin050012B001 | 42 | M | Skin | Malignant melanoma of left heel | | T3N2M1 | | - | IV | Malignant | | 2+ | 60% |
| Kin060008B002 | 52 | M | Skin | Malignant melanoma of right sole of foot | | T3N0M0 | | - | II | Malignant | | 3+ | 80% |
| Kin060015B002 | 60 | M | Skin | Malignant melanoma of right buttock | | T4N1M0 | | - | III | Malignant | | 1+ | 30% |
| Kin060033B001 | 77 | F | Skin | Malignant melanoma of left sole of foot | | T4N0M0 | | - | II | Malignant | | 0 | 0 |
| Kin060074B001 | 76 | F | Skin | Malignant melanoma of right cheek | | T3N0M0 | | - | II | Malignant | | 0 | 0 |
| Kin040074B001 | 55 | M | Skin | Malignant melanoma of right forearm | | T4N0M0 | | - | II | Malignant | | 1+ | 30% |
| Kin040075B002 | 74 | M | Skin | Malignant melanoma (sparse) of left sole of foot | | T4N0M0 | | - | II | Malignant | | 1+ | 20% |
| Kin060129B001 | 40 | M | Skin | Malignant melanoma of right sole of foot | | T3N0M0 | | - | II | Malignant | | 2+ | 50% |
| Kin040125B001 | 74 | M | Skin | Malignant melanoma of left foot | | T3aN0M0 | | - | II | Malignant | | 1+ | 20% |
| Kin030086B001 | 52 | M | Skin | Malignant melanoma (sparse) with hemorrhage and necrosis of crissum | | T3N0M0 | | - | II | Malignant | | 0 | 0 |
| Kin060017B001 | 40 | M | Skin | Malignant melanoma of right chest wall | | T4N0M0 | | - | II | Malignant | | 0 | 0 |
| Kin060195B001 | 40 | M | Skin | Malignant melanoma of back | | T4N0M0 | | - | II | Malignant | | 1+ | 20% |
| Kin060135B001 | 74 | F | Skin | Malignant melanoma of left heel | | T2N0M0 | | - | I | Malignant | | 0 | 0 |
| Kin040050B001 | 65 | M | Skin | Malignant melanoma of right thumb | | T4N0M0 | | - | II | Malignant | | 1+ | 10% |
| Kin060143B001 | 41 | M | Skin | Malignant melanoma of left leg | | T3N0M0 | | - | II | Malignant | | 1+ | 10% |
| Kin040052B001 | 76 | M | Skin | Malignant melanoma of right medial malleolus | | T4N1M0 | | - | III | Malignant | | 0 | 0 |
| Kin040053B001 | 54 | F | Skin | Malignant melanoma of left heel | | T4N0M0 | | - | II | Malignant | | 1+ | 80% |
| Kin060181B001 | 61 | F | Skin | Malignant melanoma of left sole of foot | | T4N0M0 | | - | II | Malignant | | 2+ | 80% |
| Kin060147B001 | 57 | M | Skin | Malignant melanoma of right sole of foot | | T3N0M0 | | - | II | Malignant | | 3+ | 80% |
| Kin030039B001 | 57 | M | Skin | Malignant melanoma of right cheek | | T4N0M0 | | - | II | Malignant | | 0 | 0 |
| Kin020154B001 | 46 | F | Skin | Malignant melanoma of thigh | | T4N0M0 | | - | II | Malignant | | 3+ | 80% |
| Kin030035B002 | 82 | M | Skin | Malignant melanoma of face | | T3N0M0 | | - | II | Malignant | | 0 | 0 |
| 184634B2 | 75 | F | Skin | Malignant melanoma of left buttock | | T2N0M0 | | - | I | Malignant | | 0 | 0 |
| Sst040115B001 | 54 | M | Skin | Malignant melanoma of left forefinger | | - | | - | - | Malignant | | 0 | 0 |
| Sst030215B001 | 71 | M | Skin | Malignant melanoma of right buttock | | - | | - | - | Malignant | | 1+ | 40% |
| Sst050082B001 | 45 | M | Skin | Malignant melanoma of left thigh | | - | | - | - | Malignant | | 0 | 0 |
| Sst050141B001 | 49 | M | Skin | Malignant melanoma of left thigh | | - | | - | - | Malignant | | 0 | 0 |
| Sst060045B001 | 42 | F | Skin | Malignant melanoma of right thigh | | - | | - | - | Malignant | | 0 | 0 |
| Sst050192B001 | 67 | F | Skin | Malignant melanoma of right armpit | | - | | - | - | Malignant | | 0 | 0 |
| Srm030016B001 | 7 | M | Skin | Malignant melanoma of sacrococcygeal region | | - | | - | - | Malignant | | 0 | 0 |
| Nct020139B001 | 50 | F | Left brain | Malignant melanoma | | - | | - | - | Malignant | | 2+ | 80% |
