# Supplementary material for: N-acylsphingosine amidohydrolase 1 promotes melanoma growth and metastasis by suppressing peroxisome biogenesis-induced ROS production
Source: Mol Metab. 2021 Mar 23;48:101217. doi: 10.1016/j.molmet.2021.101217 (PMC8081993; doi:10.1016/j.molmet.2021.101217)
Supplement: Supplementary file 4 — Multimedia component 4 [file mmc4.docx]

**Supplementary Table 3.** Summary of immunohistochemistry staining for ASAH1 in human normal skin and melanoma samples

| **TMA** | | **ME803b** | | | | | | | | | | | |
| --- | --- | --- | --- | --- | --- | --- | --- | --- | --- | --- | --- | --- | --- |
| **Staining** | | **Cytoplasmic** | | | | | | | | | | | |
| Density | | 0 | | | 1+ | | 2+ | | | | 3+ | | |
| Percentile | | < 10% | | | 11-25% | | 26-50% | | | | > 50% | | |
|  | | | | | | | | | | | | | |
| **Tissue** | **Normal skin** | | | | | | | | | | | | |
| **Tissue ID** | **Age** | **Sex** | **Organ (anatomic site)** | **Pathology diagnosis** | | **TNM** | | **Grade** | **Stage** | **Type** | | **Density** | **Percentile** |
| Fmg050306 | 40 | F | Skin | Adjacent normal skin tissue of breast | | - | | - | - | NAT | | 0 | 0 |
| Fmg050325 | 49 | F | Skin | Adjacent normal skin tissue of breast | | - | | - | - | NAT | | 0 | 0 |
| Fmg050396 | 53 | F | Skin | Adjacent normal skin tissue of breast | | - | | - | - | NAT | | 0 | 0 |
| Fmg050437 | 49 | F | Skin | Adjacent normal skin tissue of breast | | - | | - | - | NAT | | 0 | 0 |
| Fmg050439 | 47 | F | Skin | Adjacent normal skin tissue of breast | | - | | - | - | NAT | | 0 | 0 |
| Fmg050440 | 44 | F | Skin | Adjacent normal skin tissue of breast | | - | | - | - | NAT | | 0 | 0 |
| Fmg050336 | 42 | F | Skin | Adjacent normal skin tissue of breast | | - | | - | - | NAT | | 0 | 0 |
| Fmg050268 | 48 | F | Skin | Adjacent normal skin tissue of breast | | - | | - | - | NAT | | 0 | 0 |
| Fmg050448 | 53 | F | Skin | Adjacent normal skin tissue of breast | | - | | - | - | NAT | | 0 | 0 |
| Fmg050331 | 45 | F | Skin | Adjacent normal skin tissue of breast | | - | | - | - | NAT | | 0 | 0 |
| Fmg050442 | 46 | F | Skin | Adjacent normal skin tissue of breast | | - | | - | - | NAT | | 0 | 0 |
| Fmg050286 | 51 | F | Skin | Adjacent normal skin tissue of breast | | - | | - | - | NAT | | 0 | 0 |
| Fmg050303 | 58 | F | Skin | Adjacent normal skin tissue of breast | | - | | - | - | NAT | | 0 | 0 |
| Fmg050446 | 52 | F | Skin | Adjacent normal skin tissue of breast | | - | | - | - | NAT | | 0 | 0 |
| Fmg050441 | 43 | F | Skin | Adjacent normal skin tissue of breast | | - | | - | - | NAT | | 0 | 0 |
| Fmg050374 | 49 | F | Skin | Adjacent normal skin tissue of breast | | - | | - | - | NAT | | 0 | 0 |
| Fmg050297 | 70 | F | Skin | Adjacent normal skin tissue of breast | | - | | - | - | NAT | | 0 | 0 |
| Fmg050301 | 58 | F | Skin | Adjacent normal skin tissue of breast | | - | | - | - | NAT | | 0 | 0 |
| Fmg050483 | 46 | F | Skin | Adjacent normal skin tissue of breast | | - | | - | - | NAT | | 0 | 0 |
| Fmg050484 | 44 | F | Skin | Adjacent normal skin tissue of breast | | - | | - | - | NAT | | 0 | 0 |
| Fmg050472 | 35 | F | Skin | Adjacent normal skin tissue of breast | | - | | - | - | NAT | | 0 | 0 |
| Fmg050477 | 42 | F | Skin | Adjacent normal skin tissue of breast | | - | | - | - | NAT | | 0 | 0 |
| Kin040162 | 42 | F | Skin | Adjacent normal skin tissue of abdominal wall | | - | | - | - | NAT | | 0 | 0 |
| Kin040180 | 50 | F | Skin | Adjacent normal skin tissue of breast | | - | | - | - | NAT | | 0 | 0 |
| Kin060181 | 61 | F | Skin | Adjacent normal skin tissue of left sole of foot | | - | | - | - | NAT | | 0 | 0 |
| Kin060081 | 45 | F | Skin | Adjacent normal skin tissue of eyebrow | | - | | - | - | NAT | | 0 | 0 |
| Kin030264 | 31 | M | Skin | Adjacent normal skin tissue of thigh | | - | | - | - | NAT | | 0 | 0 |
| Kin050039 | 51 | M | Skin | Adjacent normal skin tissue of left upper abdominal wall | | - | | - | - | NAT | | 0 | 0 |
| Kin030295 | 75 | M | Skin | Adjacent normal skin tissue of left leg | | - | | - | - | NAT | | 0 | 0 |
| Kin050052 | 57 | F | Skin | Adjacent normal skin tissue of scalp | | - | | - | - | NAT | | 0 | 0 |
| Kin07N028 | 37 | M | Skin | Skin tissue of abdominal part | | - | | - | - | Normal | | 0 | 0 |
| Kin07N025 | 27 | F | Skin | Skin tissue of scalp | | - | | - | - | Normal | | 0 | 0 |
| Kin07N029 | 30 | M | Skin | Skin tissue of abdominal part | | - | | - | - | Normal | | 0 | 0 |
| Kin07N026 | 33 | M | Skin | Skin tissue of abdominal part | | - | | - | - | Normal | | 0 | 0 |
| Kin07N017 | 50 | M | Skin | Skin tissue of scalp | | - | | - | - | Normal | | 0 | 0 |
| Kin08N033 | 42 | F | Skin | Skin tissue of abdominal part | | - | | - | - | Normal | | 0 | 0 |
| Kin06N005 | 47 | M | Skin | Skin tissue of wrist | | - | | - | - | Normal | | 0 | 0 |
| Kin08N001 | 35 | M | Skin | Skin tissue | | - | | - | - | Normal | | 0 | 0 |
| Kin08N003 | 40 | M | Skin | Skin tissue | | - | | - | - | Normal | | 0 | 0 |
| Kin08N004 | 30 | M | Skin | Skin tissue of wrist | | - | | - | - | Normal | | 0 | 0 |
|  | | | | | | | | | | | | | |
| **Tissue** | **Melanoma** | | | | | | | | | | | | |
| **Tissue ID** | **Age** | **Sex** | **Organ (anatomic site)** | **Pathology diagnosis** | | **TNM** | | **Grade** | **Stage** | **Type** | | **Density** | **Percentile** |
| Fvl041282 | 41 | F | Vulva | Malignant melanoma | | T4N0M0 | | - | II | Malignant | | 0 | 0 |
| Fvl050429 | 38 | F | Vulva | Malignant melanoma | | T4N0M0 | | - | II | Malignant | | 2 | > 75 |
| Fvl032365 | 45 | F | Vulva | Malignant melanoma | | T4N0M0 | | - | II | Malignant | | 1 | > 75 |
| Fvl060506 | 79 | F | Vulva | Malignant melanoma of vagina | | - | | - | - | Malignant | | 2 | > 75 |
| Fvl041203 | 62 | F | Vulva | Malignant melanoma of vagina | | - | | - | - | Malignant | | 3 | > 75 |
| Fvl041118 | 34 | F | Vulva | Malignant melanoma | | T4N0M0 | | - | II | Malignant | | 1 | > 75 |
| Fvl040938 | 15 | F | Vulva | Malignant melanoma of clitoris | | T4N0M0 | | - | II | Malignant | | 1 | > 75 |
| Fvl030086 | 57 | F | Vulva | Malignant melanoma | | T4N0M0 | | - | II | Malignant | | 1 | > 75 |
| Dre041810 | 72 | F | Rectum | Malignant melanoma | | - | | - | - | Malignant | | 1 | > 75 |
| Dre061446 | 57 | M | Rectum | Malignant melanoma | | - | | - | - | Malignant | | 2 | > 75 |
| Dre061865 | 52 | F | Rectum | Malignant melanoma of anal canal | | - | | - | - | Malignant | | 1 | > 75 |
| Dre062316 | 47 | M | Rectum | Malignant melanoma | | - | | - | - | Malignant | | 1 | > 75 |
| Dre031701 | 84 | F | Rectum | Malignant melanoma of crissum | | - | | - | - | Malignant | | 0 | 0 |
| Dre023410 | 64 | F | Rectum | Malignant melanoma | | - | | - | - | Malignant | | 1 | > 75 |
| Dre062768 | 67 | M | Rectum | Malignant melanoma | | - | | - | - | Malignant | | 5 | > 75 |
| Dre061890 | 42 | F | Rectum | Malignant melanoma | | - | | - | - | Malignant | | 3 | > 75 |
| Dre010332 | 38 | F | Rectum | Malignant melanoma | | - | | - | - | Malignant | | 1 | > 75 |
| Din024558 | 71 | M | Small intestine | Malignant melanoma | | - | | - | - | Malignant | | 2 | > 75 |
| Des050286 | 64 | M | Esophagus | Malignant melanoma | | - | | - | - | Malignant | | 3 | > 75 |
| Dre030138 | 67 | F | Rectum | Malignant melanoma | | - | | - | - | Malignant | | 0 | 0 |
| Dre080282 | 66 | F | Rectum | Malignant melanoma | | - | | - | - | Malignant | | 1 | > 75 |
| 181422 | 60 | F | Mandible | Malignant melanoma | | - | | - | - | Malignant | | 3 | > 75 |
| 181423 | 60 | F | Mandible | Malignant melanoma | | - | | - | - | Malignant | | 2 | > 75 |
| Atc060179 | 40 | M | Skin | Malignant melanoma of right chest wall | | T4N0M0 | | - | II | Malignant | | 3 | > 75 |
| Kin020178 | 66 | M | Skin | Malignant melanoma of right thigh | | T4N0M0 | | - | II | Malignant | | 3 | > 75 |
| Kin060042 | 56 | F | Skin | Malignant melanoma of left leg | | T3N0M0 | | - | II | Malignant | | 3 | > 75 |
| Kin060059 | 36 | M | Skin | Malignant melanoma of right chest wall | | T4N0M0 | | - | II | Malignant | | 0 | 0 |
| Kin060158 | 52 | M | Skin | Malignant melanoma of left lower abdominal wall | | T4N0M0 | | - | II | Malignant | | 1 | > 75 |
| Kin060160 | 52 | F | Skin | Malignant melanoma of left heel | | T3N0M0 | | - | II | Malignant | | 1 | > 75 |
| Kin030099 | 47 | F | Skin | Malignant melanoma of right upper arm | | T2N0M0 | | - | IB | Malignant | | 1 | > 75 |
| Kin060124 | 80 | M | Skin | Malignant melanoma of right sole of foot | | T4N0M0 | | - | II | Malignant | | 2 | > 75 |
| Kin060135 | 74 | F | Skin | Malignant melanoma of left heel | | T2N0M0 | | - | IB | Malignant | | 0 | 0 |
| Kin060143 | 41 | M | Skin | Malignant melanoma of left leg | | T3N0M0 | | - | II | Malignant | | 3 | > 75 |
| Kin060181 | 41 | M | Skin | Malignant melanoma of left sole of foot | | T4N0M0 | | - | II | Malignant | | 1 | > 75 |
| Kin060147 | 57 | M | Skin | Malignant melanoma of right sole of foot | | T3N0M0 | | - | II | Malignant | | 3 | > 75 |
| Kin060195 | 40 | M | Skin | Malignant melanoma of back | | T4N0M0 | | - | II | Malignant | | 1 | > 75 |
| Kin060148 | 51 | M | Skin | Malignant melanoma of chest wall | | T4N0M0 | | - | II | Malignant | | 0 | 0 |
