# Supplementary material for: N-acylsphingosine amidohydrolase 1 promotes melanoma growth and metastasis by suppressing peroxisome biogenesis-induced ROS production
Source: Mol Metab. 2021 Mar 23;48:101217. doi: 10.1016/j.molmet.2021.101217 (PMC8081993; doi:10.1016/j.molmet.2021.101217)
Supplement: Supplementary file 5 — Multimedia component 5 [file mmc5.docx]

**Supplementary Table 4.** Analysis of transcription factors on *ASAH1* promoter by PROMO 3.0 and rVISTA 2.0

| **S.No.** | **Transcription factor** | | **Predicted binding site on *ASAH1* promoter (5’-3’ upstream of transcription start)** | **Consensus DNA-binding sequence (5’-3’)** |
| --- | --- | --- | --- | --- |
| 1 | AhR:Arnt | ^-695^ GGCAGCGTGC ^-686^ | | GCACGCCAGC |
| 2 | AP-1 | ^-225^ TGACTCCGC ^-217^ | | GATGAGTCA |
| 3 | AP-2alphaA | ^-68^ GCCTGC ^-63^ | | GCAGGC |
| 4 | AR | ^-1259^ TATCTGTCC ^-1251^ | | GCACTGTCC |
| 5 | ATF1 | ^-650^ AGACGTCAGAGG ^-639^ | | TGACGTCATGGG |
| 6 | ATF3 | ^-650^ AGACGTCA ^-643^ | | TGACGTAA |
| 7 | C/EBPalpha | ^-344^ CTCAATT ^-338^ | | GATTGAG |
| 8 | C/EBPbeta | ^-321^ TTGC ^-318^  ^-1129^ TTGC ^-1126^  ^-1500^ TTGC ^-1497^  ^-1775^ TTGC ^-1772^ | | TTGC |
| 9 | c-Myb | ^-458^ GCCAGTTA ^-451^ | | CAACTGCC |
| 10 | CREB | ^-651^ GAGACGTCA ^-643^ | | CTGACGTCA |
| 11 | E2F1 | ^-86^ GCGGGACA ^-79^ | | TTTCCCGC |
| 12 | EBF | ^-1103^ GCCTCAGGGTG ^-1093^ | | CCCCCAGGGCA |
| 13 | ELF-1 | ^-635^ ATCCAGGAAGTGC ^-623^ | | CCACTTCCTAGAG |
| 14 | Elk-1 | ^-1311^ CTTCCTGGT ^-1303^ | | CTTCCTCCC |
| 15 | ETF | ^-23^ GCCCCACCTAC ^-13^ | | GCCCCCCGCAC |
| 16 | FOXP3 | ^-1642^ AACAAC ^-1637^ | | AACAAC |
| 17 | GATA-1 | ^-1259^ TATCTG ^-1254^  ^-1896^ TATCTG ^-1891^ | | TATCTG |
| 18 | GR | ^-728^ CAAAAAG ^-722^  ^-804^ CAAAAAG ^-798^ | | CAAAAAA |
| 19 | IRF-1 | ^-1739^ GTAGGGAAA ^-1731^ | | AAAGGGAAA |
| 20 | LEF-1 | ^-678^ CATCAAAG ^-671^ | | CTTTGATC |
| 21 | NF-1 | ^-1034^ TTGGCACA ^-1027^ | | TTGGCCCA |
| 22 | NF-Y | ^-1759^ TCACCAAT ^-1752^ | | ATTGGTCA |
| 23 | p53 | ^-614^ GGGCAGG ^-608^ | | CATGCCC |
| 24 | PU-1 | ^-635^ ATCCAGGAAGTGC ^-623^ | | ACACTTCCTCTAG |
| 25 | RBP-Jkappa | ^-1633^ AAGATGGGAATC ^-1622^ | | GTTTCCCACGAC |
| 26 | SRY | ^-679^ TCATCAAAG ^-671^ | | AGAACAAAG |
| 27 | STAT4 | ^-607^ ATTTCC ^-602^  ^-820^ ATTTCC ^-815^ | | ATTTCC |
| 28 | STAT5A | ^-183^ GCTTTTCTCAGAG ^-171^ | | TTACCAGAAAAGG |
| 29 | TCF-4 | ^-1863^ CCTTTGAAAG ^-1854^ | | CCTTTGAAAG |
| 30 | VDR | ^-1068^ GTTCACTGC ^-1060^ | | CGGGTGAAC |
| 31 | XBP-1 | ^-1514^ ATGCCT ^-1509^ | | ATGACG |
| 32 | YY1 | ^-52^ CCAT ^-49^  ^-978^ CCAT ^-975^  ^-1125^ CCAT ^-1122^  ^-1380^ CCAT ^-1377^  ^-1583^ CCAT ^-1580^  ^-1792^ CCAT ^-1789^ | | CCAT |
